# Supplementary material for: Space-time smoothing of mortality estimates in children aged 5-14 in Sub-Saharan Africa
Source: PLoS One. 2021 Jan 19;16(1):e0245596. doi: 10.1371/journal.pone.0245596 (PMC7815126; doi:10.1371/journal.pone.0245596)
Supplement: S1 Appendix — (PDF) [file pone.0245596.s001.pdf]

S1 Appendix for "Space-time smoothing of mortality estimates in children aged 5-14 in Sub-Saharan Africa"

Benjamin-Samuel Schluter[1*], Bruno Masquelier[1]

1 DEMO, University of Louvain (UCLouvain), Louvain-la-Neuve, Belgium
* benjamin-samuel.schluter@uclouvain.be

December 22, 2020

Contents

S1 List of DHS surveys included in the analysis **3**

S2 Definition of cut points for periods **5**

S3 Reduction in uncertainty in sub-national estimates of $_{10}q_5$ through space-time smoothing **6**

S4 Comparison between national-level model and UN IGME estimates of $_{10}q_5$ **17**

S5 Sub-national mortality rates **22**

S6 Assessment of precision of mortality estimates for children aged less than five years **33**

S7 Assessment of precision of mortality estimates for children aged 5-14 with a 15% threshold **34**

S8 Mortality estimates in our sample, compared to all countries in Sub-Saharan Africa **35**

S1 List of DHS surveys included in the analysis

Table S1. DHS surveys, sample sizes and number of Admin-1 areas

Country	Year	Sample size (women 15-49)	Admin 1 units
Benin	1996	5,491	6
	2001	5,769	
	2006	17,794	
	2012	16,599	
	2018	15,928	
Burkina Faso	1993	6,354	5
	1999	6,445	
	2003	12,477	
	2010	17,087	
Cameroon	1998	5,501	5
	2004	10,656	
	2011	15,426	
	2018	13,527	
Ethiopia	2000	15,367	11
	2005	14,070	
	2011	16,515	
	2016	15,683	
Ghana	1993	4,562	10
	1998	4,843	
	2003	5,691	
	2008	4,916	
	2014	9,396	
Guinea	1999	6,753	5
	2005	7,954	
	2012	9,142	
	2018	8,000	
Kenya	1993	7,540	8
	1998	7,881	
	2003	8,195	
	2008	8,444	
	2014	31,079	
Lesotho	2004	7,095	10
	2009	7,624	
	2014	6,621	
Madagascar	1992	6,260	6
	1997	7,060	
	2004	7,949	
	2009	17,375	

Country	Year	Sample size (women 15-49)	Admin 1 units
Malawi	1992	4,849	3
	2000	13,220	
	2004	11,698	
	2010	23,020	
	2016	24,562	
Mali	1996	9,704	7
	2001	12,849	
	2006	14,583	
	2013	10,424	
	2018	10,519	
Namibia	2000	6,755	13
	2007	9,804	
	2013	9,176	
Niger	1992	6,503	6
	1998	7,577	
	2006	9,223	
	2012	11,160	
Nigeria	1990	8,781	6
	2003	7,620	
	2008	33,385	
	2013	38,948	
	2018	41,821	
Rwanda	2005	11,321	5
	2008	7,313	
	2010	13,671	
	2015	13,497	
Senegal	1993	6,310	14
	1997	8,593	
	2005	14,602	
	2011	15,688	
	2013	8,636	
	2014	8,488	
	2015	8,851	
	2016	8,865	
	2017	16,787	
	2018	9,414	
	2019	8,649	
Tanzania	1996	8,120	6
	1999	4,029	
	2005	10,329	
	2010	10,139	
	2016	13,266	

Country	Year	Sample size (women 15-49)	Admin 1 units
Uganda	1995	7,070	4
	2001	7,246	
	2006	8,531	
	2011	8,674	
	2016	18,506	
Zambia	1992	7,060	9
	1996	8,021	
	2002	7,658	
	2007	7,146	
	2014	16,411	
	2018	13,683	
Zimbabwe	1994	6,128	10
	1999	5,907	
	2006	8,907	
	2011	9,171	
	2015	9,955	

S2 Definition of cut points for periods

Because we need to combine surveys whose fieldwork dates have varied over time, we let the most recent survey defines the time breaks. If the fieldwork for the most recent survey for a particular country ended before September, the upper bound of the selected period was the last day of the preceding year (December 31). Otherwise, it was set to December 31 of the year of the fieldwork. This avoided having no observation for more than a quarter of a year for most recent estimates.

We illustrate below how time periods were defined to pool together multiple datasets, using Nigeria as an example (Fig S1).

Fig S1. Cut points for Nigeria

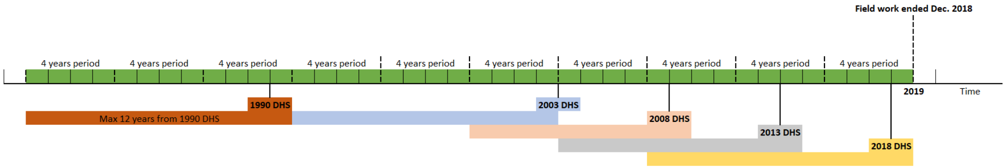

The fieldwork of the most recent DHS in Nigeria ended in December 2018. As December comes after September, our upper bound consists of the year 2019. Our most recent period is thus [2015, 2018]. From there, we define a series of 4 years period going backward in time: [2011, 2014], [2007, 2010], [2003, 2006], [1999, 2002], [1995, 1998], [1991, 1994], [1987, 1990], [1983, 1986] and [1979, 1982]. The lower bound of the oldest period has to be at most 12 years from the oldest survey (i.e 1990 DHS). This consists of the 4 years period series for Nigeria. Then, each DHS survey is used to obtain estimates for specific periods according to when the survey took place. More specifically, 2018 DHS is used to inform estimates associated to three periods [2007, 2010], [2011, 2014] and [2015, 2018]. It does not inform later periods as we analyze only the period of 0-12 completed years before the survey. Looking at 2008 DHS, its field work ended in October. Hence, it consists of more than one year of observation in period [2007, 2010]

and provides information for estimates in [2007, 2010], [2003, 2006] and [1999, 2002].

We used a maximum of 24 years from a survey to obtain estimates of under-five mortality.

S3 Reduction in uncertainty in sub-national estimates of ${}_{10}q_5$ through space-time smoothing

In the figures that follow, we compare estimates and their associated uncertainty obtained after the meta-analysis step (which consists in pooling information across different DHS surveys for a given sub-national unit and period) with final estimates obtained after space-time smoothing. In some cases (e.g. North Eastern, Kenya before 1991), no estimate is presented for the meta-analysis as no DHS survey provided information on this particular combination of sub-national unit and period.

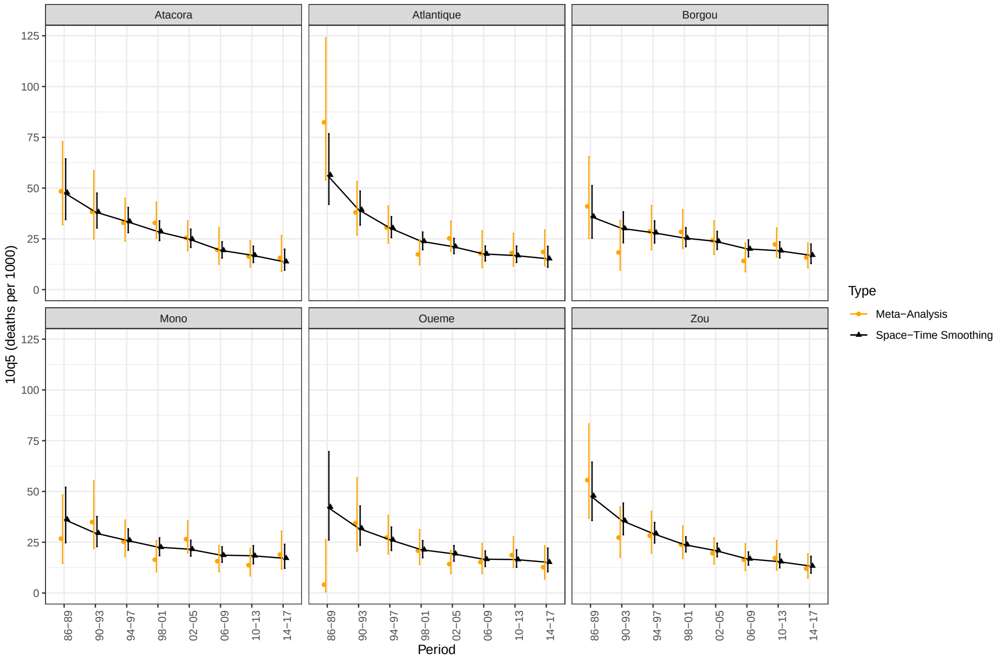

Fig S2. Meta-analysis and space-time smoothing estimates of ${}_{10}q_5$, Benin

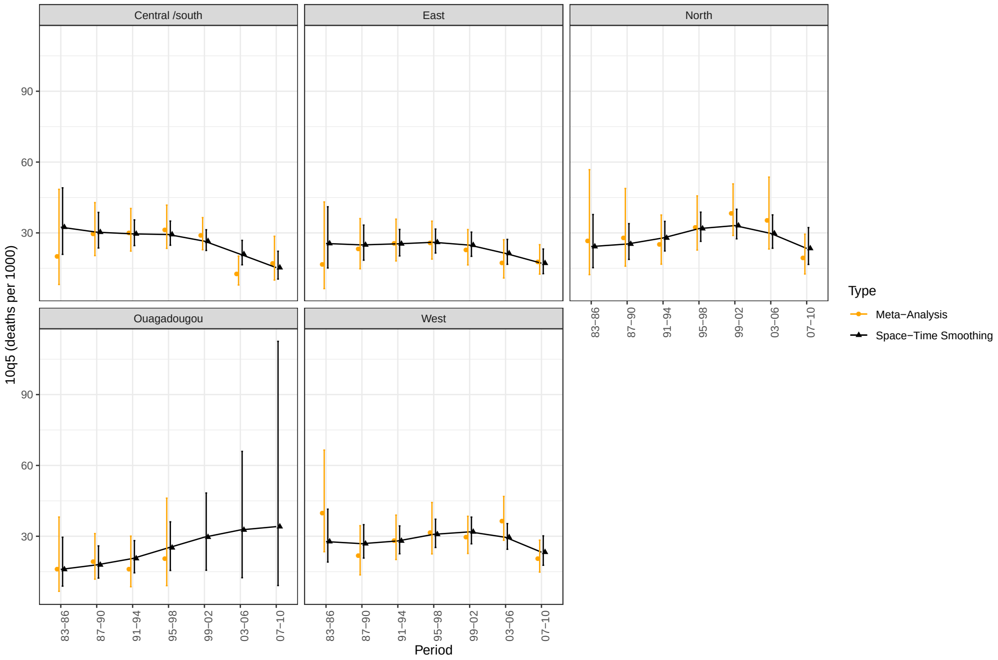

Fig S3. Meta-analysis and space-time smoothing estimates of ${}_{10}q_5$, Burkina Faso

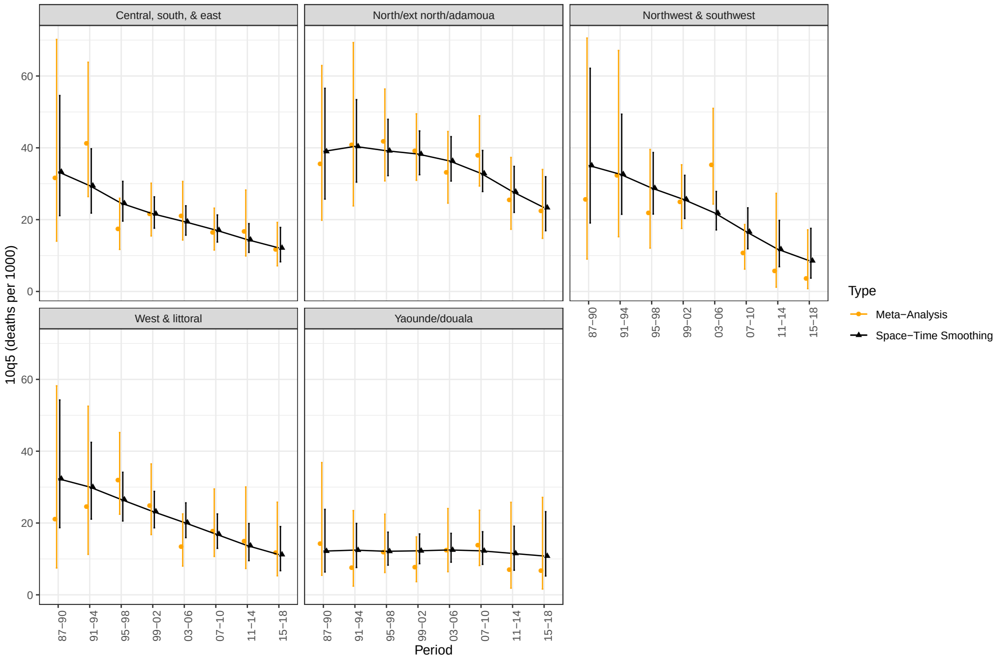

Fig S4. Meta-analysis and space-time smoothing estimates of ${}_{10}q_5$, Cameroon

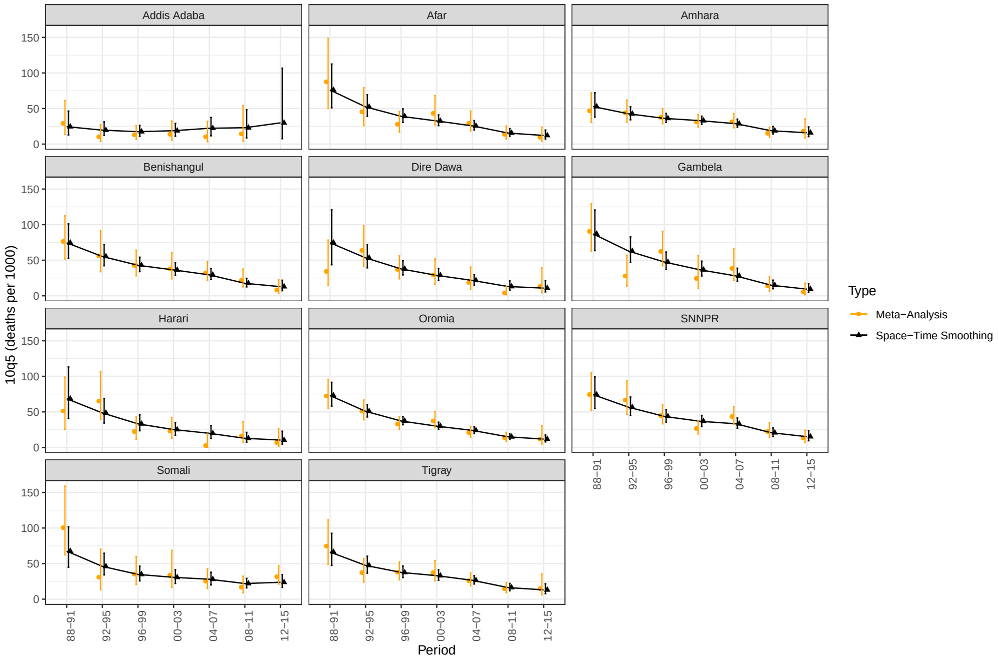

Fig S5. Meta-analysis and space-time smoothing estimates of ${}_{10}q_5$, Ethiopia

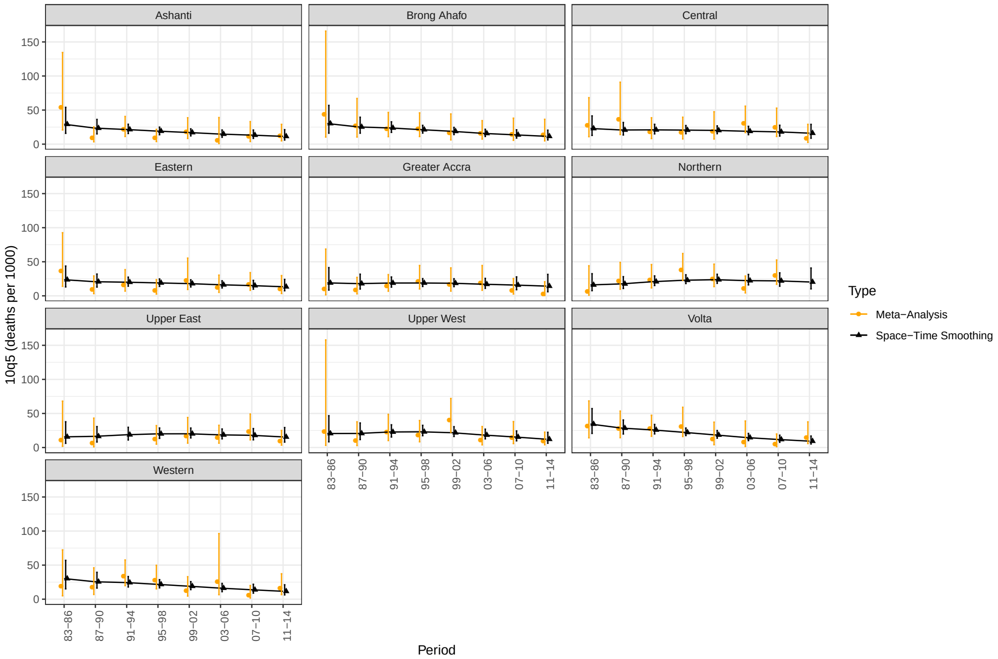

Fig S6. Meta-analysis and space-time smoothing estimates of ${}_{10}q_5$, Ghana

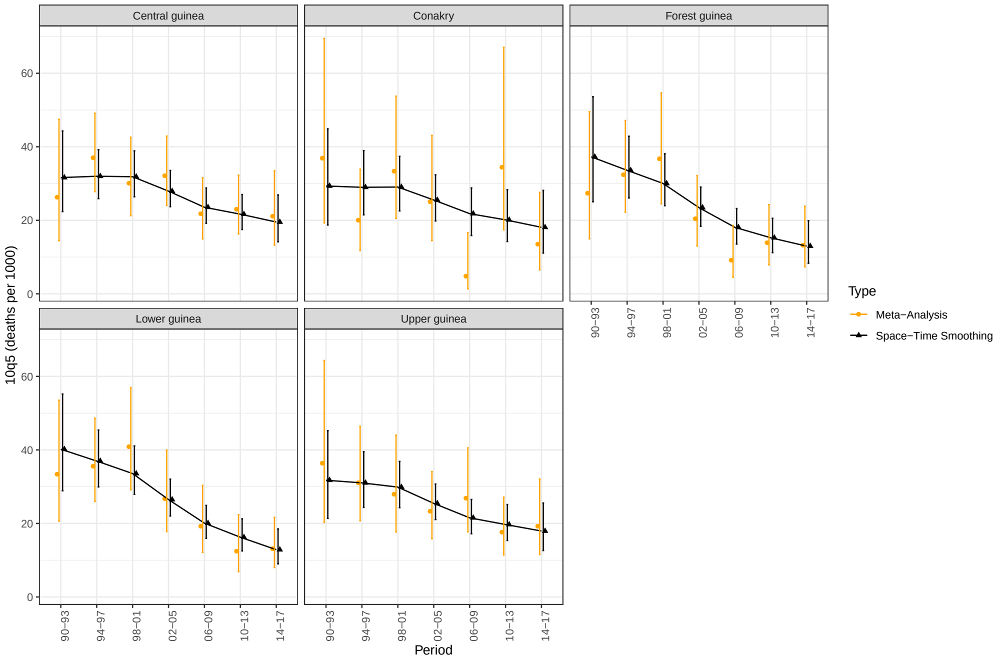

Fig S7. Meta-analysis and space-time smoothing estimates of ${}_{10}q_5$, Guinea

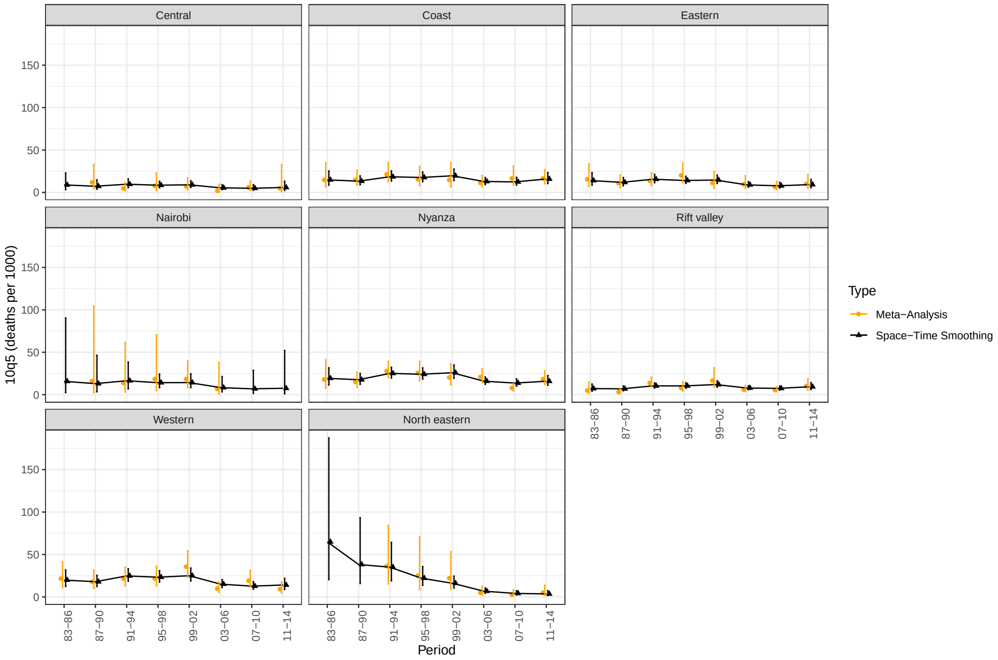

Fig S8. Meta-analysis and space-time smoothing estimates of ${}_{10}q_5$, Kenya

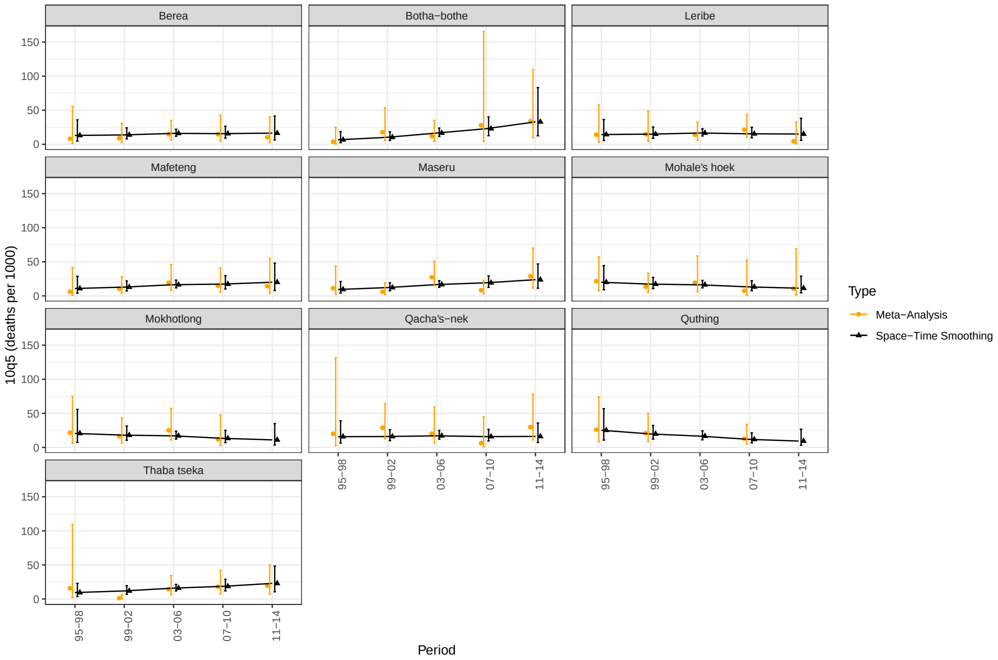

Fig S9. Meta-analysis and space-time smoothing estimates of ${}_{10}q_5$, Lesotho

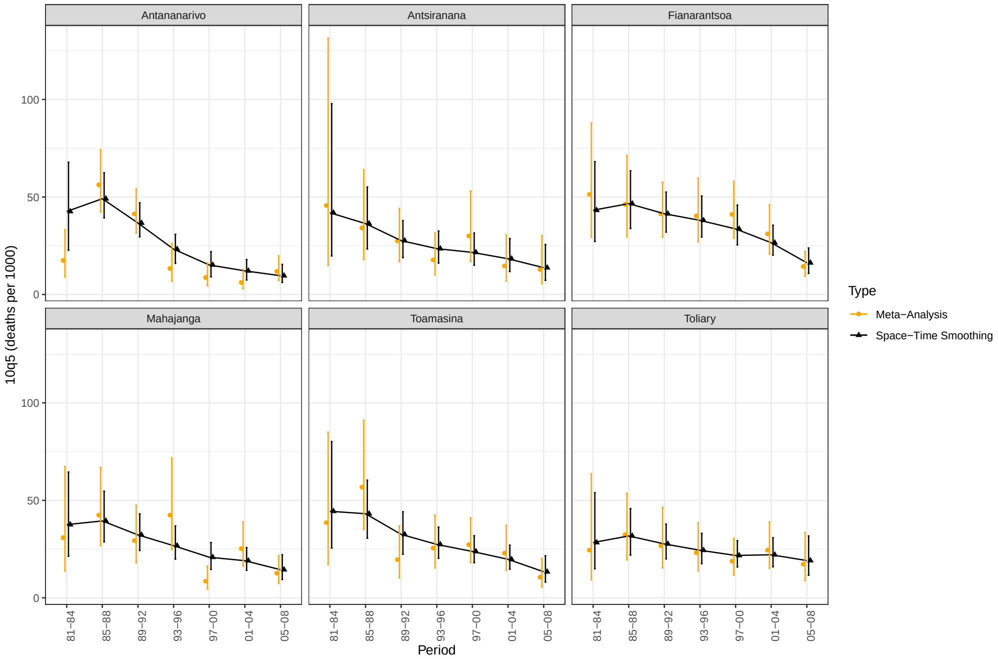

Fig S10. Meta-analysis and space-time smoothing estimates of ${}_{10}q_5$, Madagascar

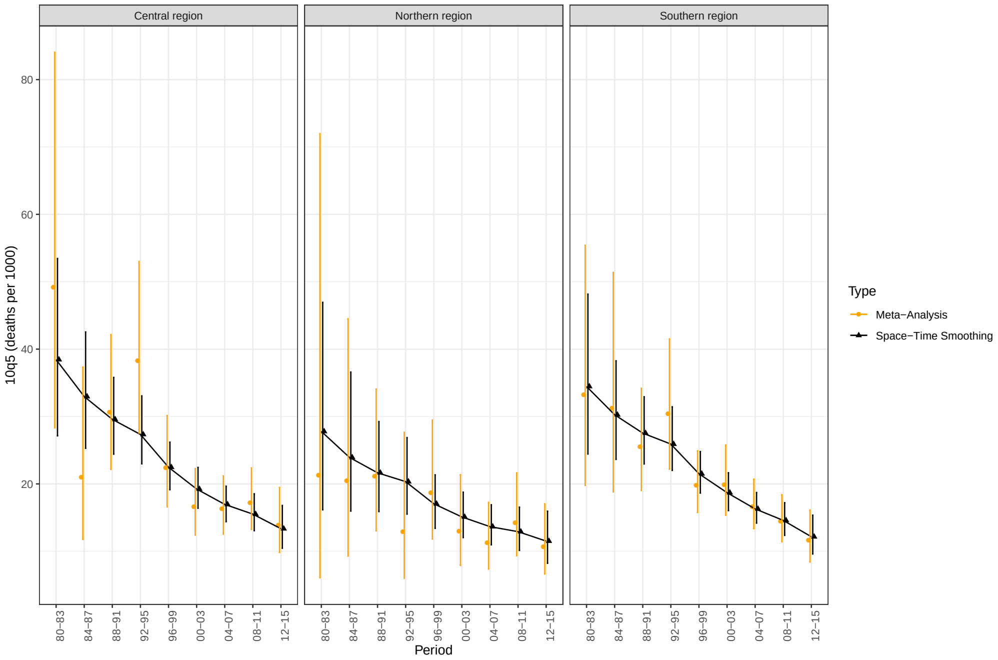

Fig S11. Meta-analysis and space-time smoothing estimates of ${}_{10}q_5$, Malawi

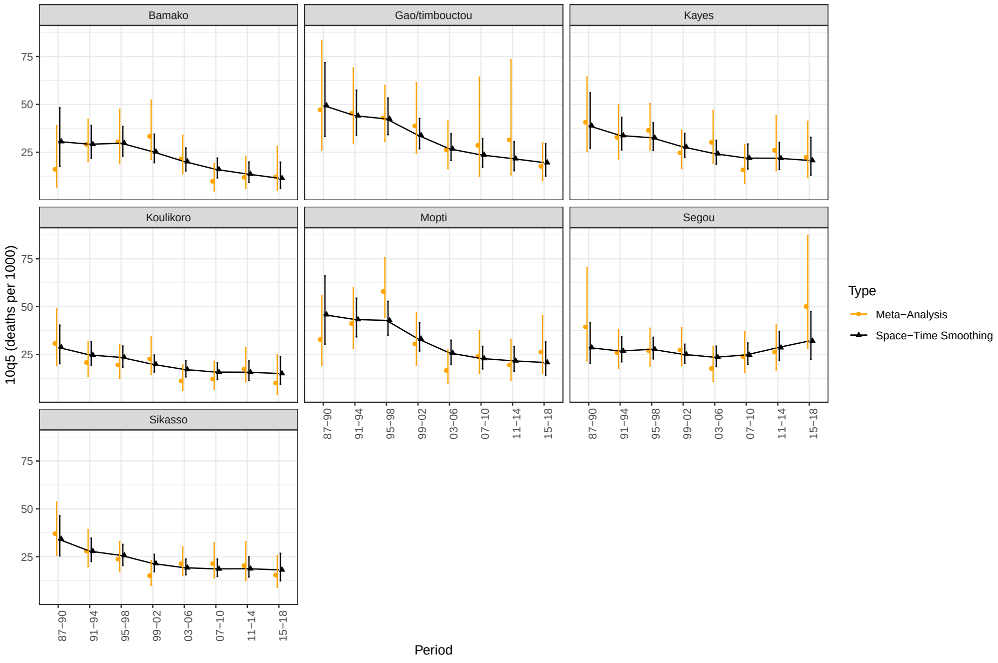

Fig S12. Meta-analysis and space-time smoothing estimates of ${}_{10}q_5$, Mali

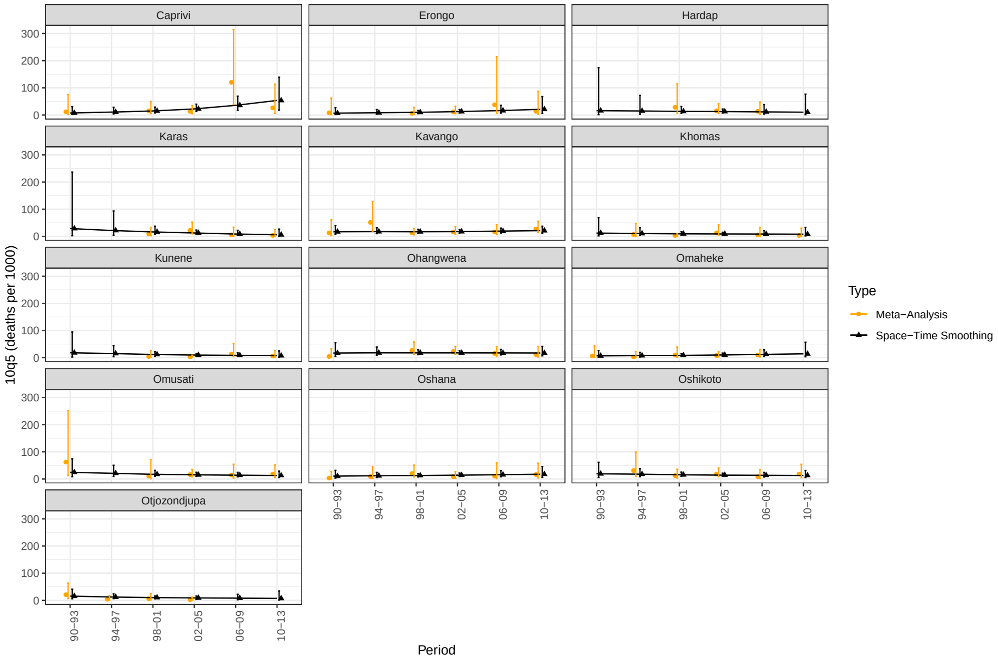

Fig S13. Meta-analysis and space-time smoothing estimates of ${}_{10}q_5$, Namibia

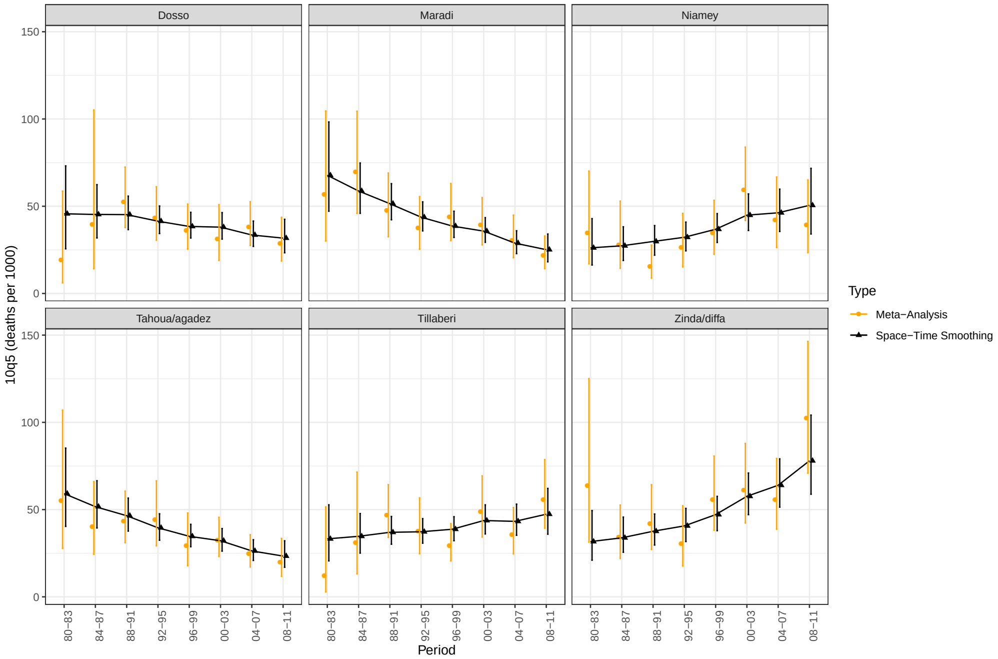

Fig S14. Meta-analysis and space-time smoothing estimates of ${}_{10}q_5$, Niger

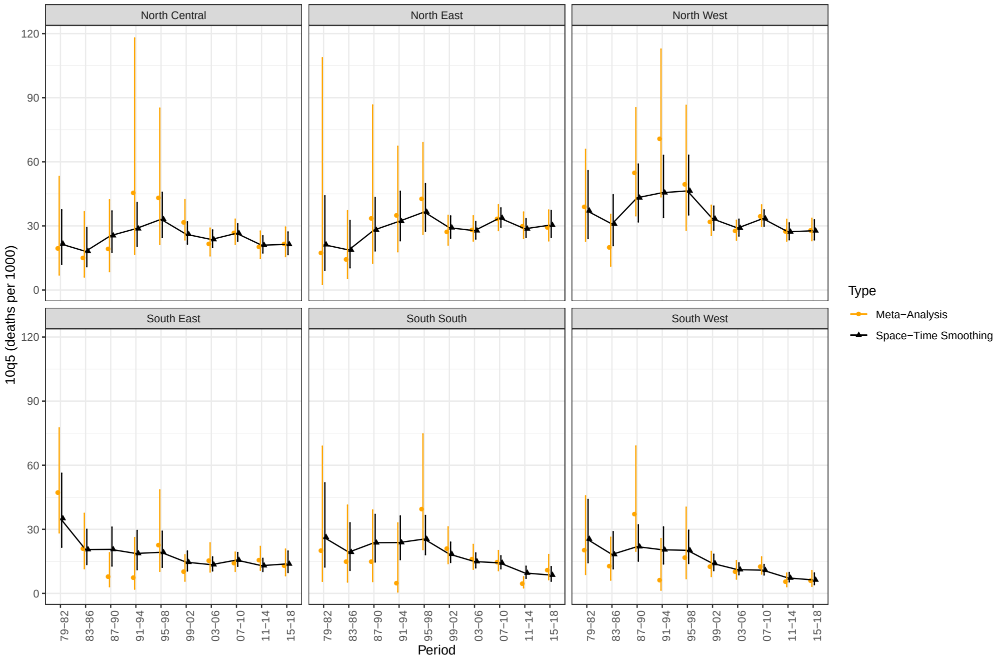

Fig S15. Meta-analysis and space-time smoothing estimates of $_{10}q_5$, Nigeria

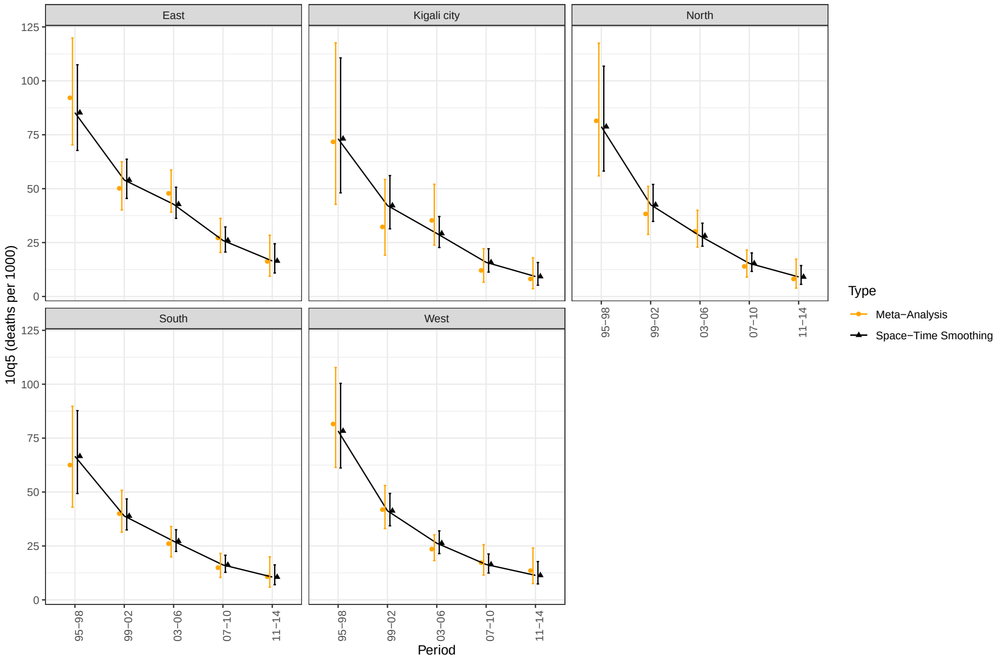

Fig S16. Meta-analysis and space-time smoothing estimates of $_{10}q_5$, Rwanda

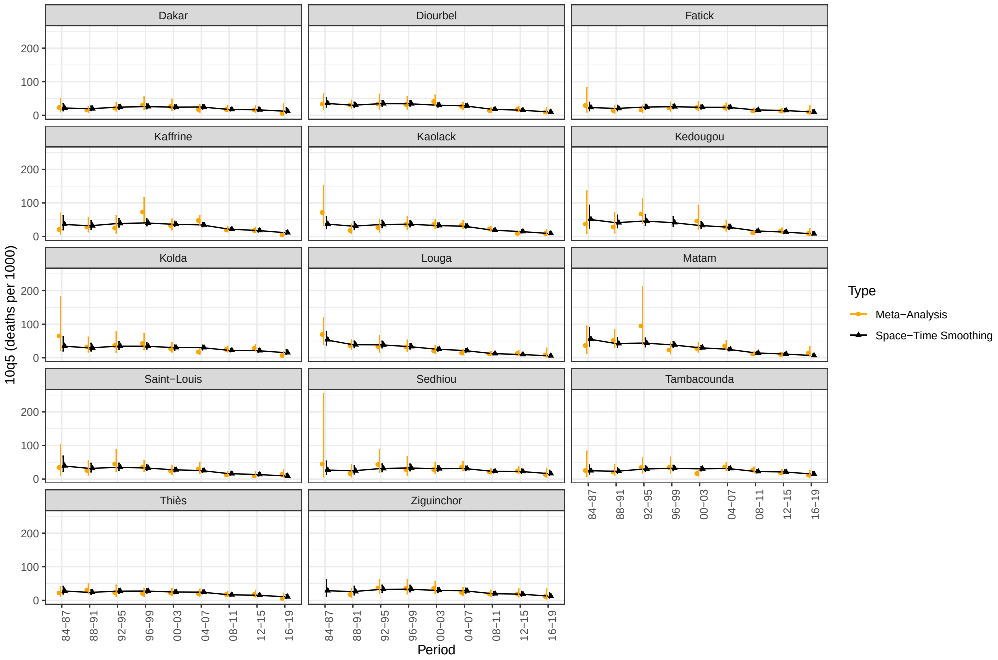

Fig S17. Meta-analysis and space-time smoothing estimates of ${}_{10}q_5$, Senegal

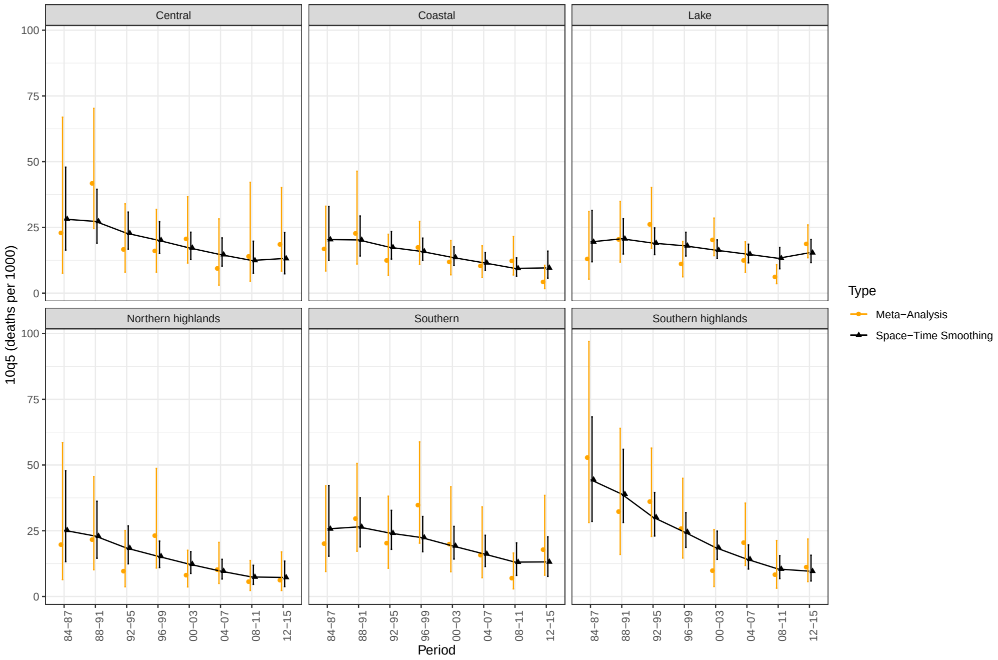

Fig S18. Meta-analysis and space-time smoothing estimates of ${}_{10}q_5$, Tanzania

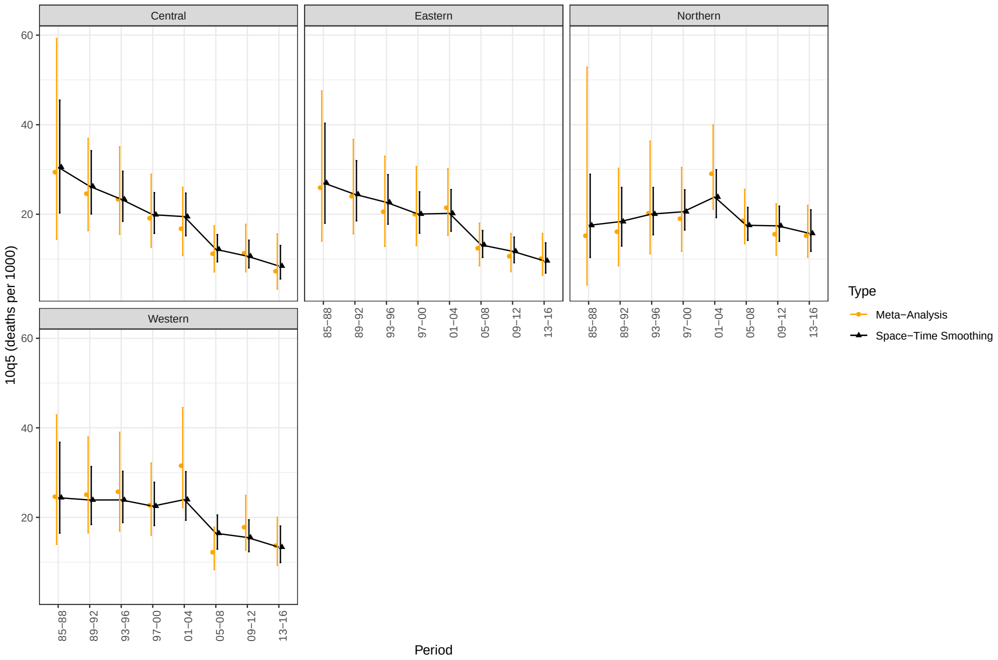

Fig S19. Meta-analysis and space-time smoothing estimates of ${}_{10}q_5$, Uganda

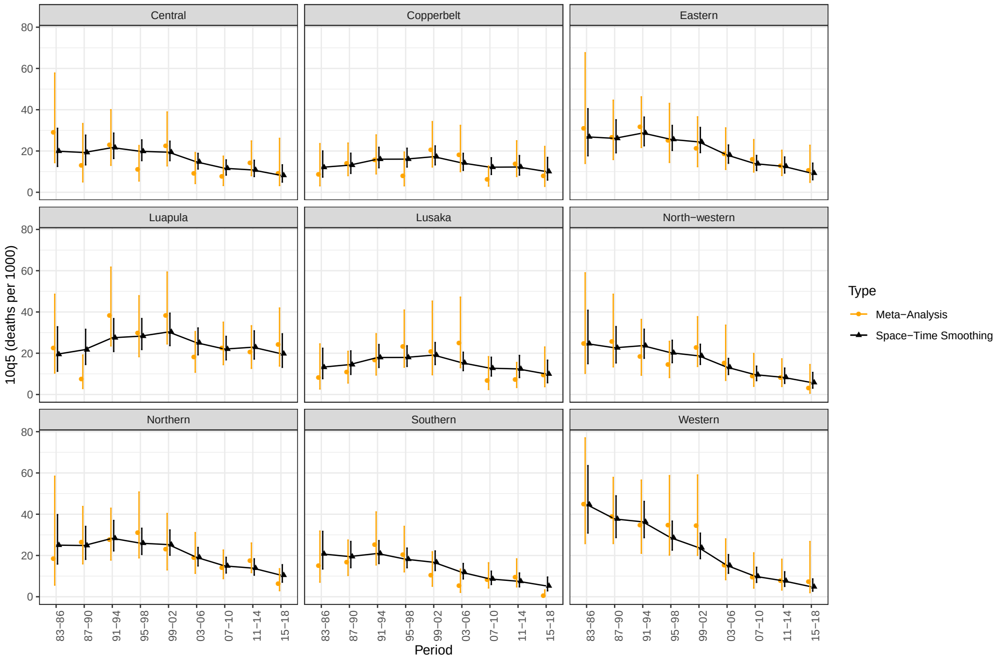

Fig S20. Meta-analysis and space-time smoothing estimates of ${}_{10}q_5$, Zambia

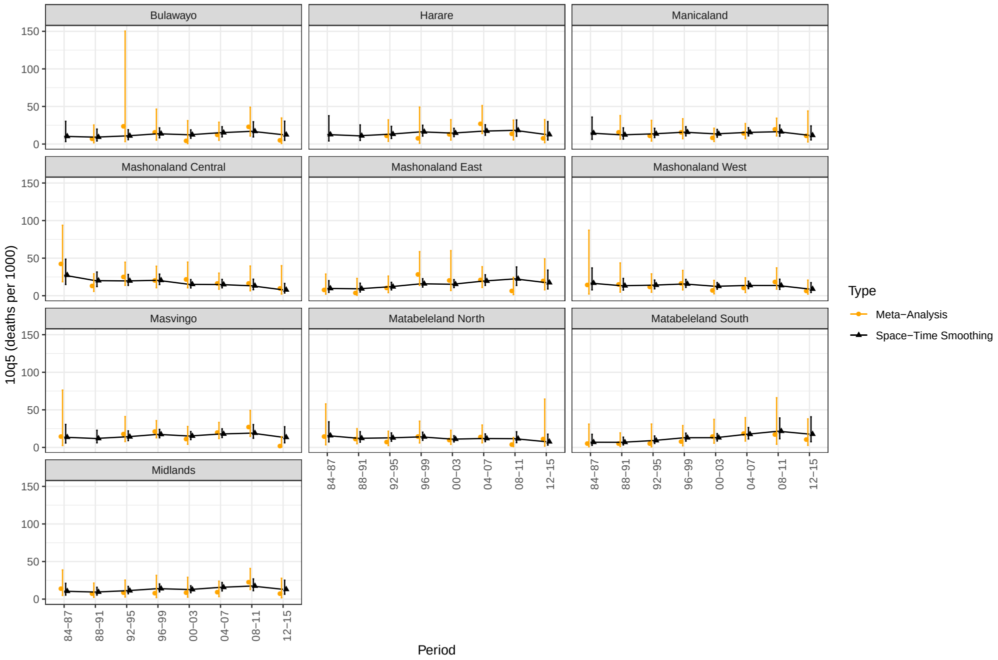

Fig S21. Meta-analysis and space-time smoothing estimates of ${}_{10}q_5$, Zimbabwe

S4 Comparison between national-level model and UN IGME estimates of ${}_{10}q_5$

In this section, we compare the national estimates of the probability ${}_{10}q_5$ that can be obtained by pooling surveys together with those estimated by the UN IGME, based on the B3 model [1,2]. The national model is also a meta-analysis of direct national estimates (where sampling weights are taken into account in a logistic regression), where the weight depends on the inverse of the variance of the national estimates from different surveys. After this meta-analysis, there is a simple temporal smoothing (RW2), without spatial smoothing [3]. The UN IGME estimates are based in part on the same DHS surveys, but are also informed by other data series, such as MICS surveys or reports from censuses on recent household deaths. In addition, the B3 model used for reconstructing trends in mortality includes a data model that captures recall and truncation biases. There is evidence that DHS surveys tend to slightly under-estimate mortality in the age group 5-14, when compared to other data series [4]. As a result, the final estimates are adjusted upwards and may fall above the original survey data points. This explains why the UN IGME estimates tend to be higher than our estimates from the national-level model, based solely on DHS without adjustment for non-sampling errors.

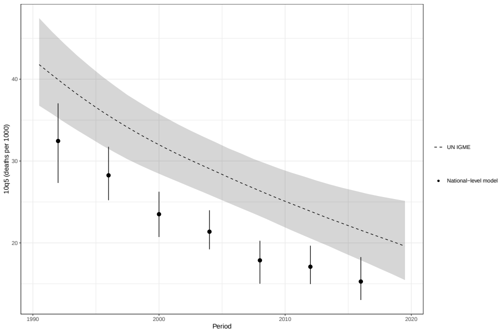

Fig S22. National-level model and UN IGME ${}_{10}q_5$ estimates, Benin

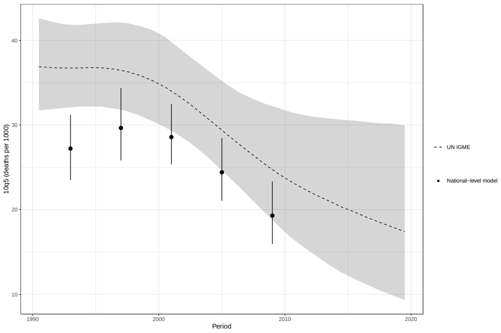

Fig S23. National-level model and UN IGME ${}_{10}q_5$ estimates, Burkina Faso

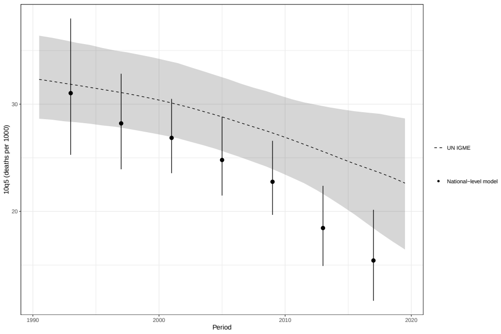

Fig S24. National-level model and UN IGME ${}_{10}q_5$ estimates, Cameroon

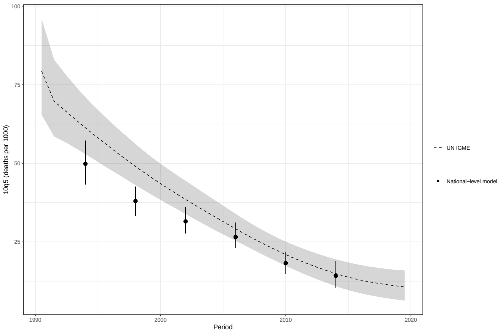

Fig S25. National-level model and UN IGME ${}_{10}q_5$ estimates, Ethiopia

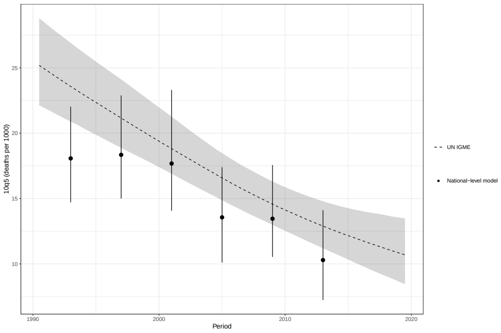

Fig S26. National-level model and UN IGME ${}_{10}q_5$ estimates, Ghana

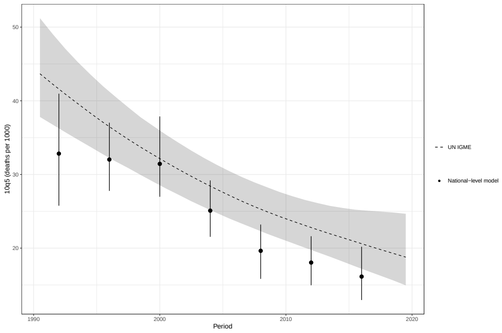

Fig S27. National-level model and UN IGME ${}_{10}q_5$ estimates, Guinea

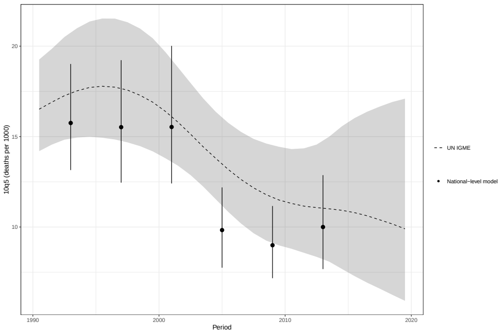

Fig S28. National-level model and UN IGME ${}_{10}q_5$ estimates, Kenya

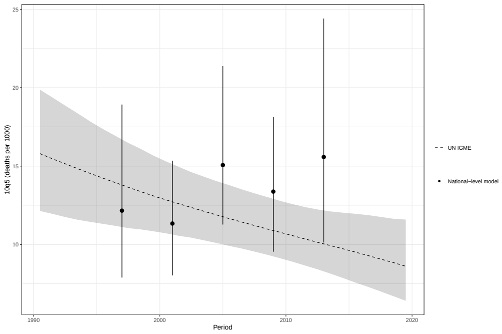

Fig S29. National-level model and UN IGME ${}_{10}q_5$ estimates, Lesotho

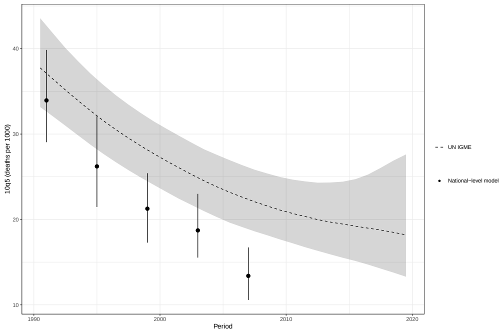

Fig S30. National-level model and UN IGME ${}_{10}q_5$ estimates, Madagascar

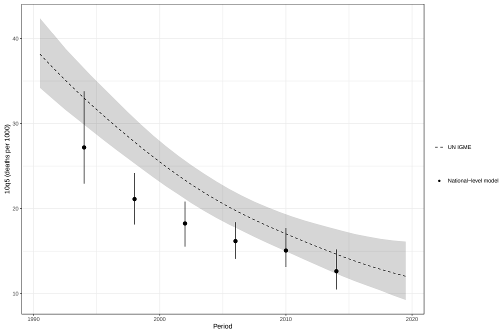

Fig S31. National-level model and UN IGME ${}_{10}q_5$ estimates, Malawi

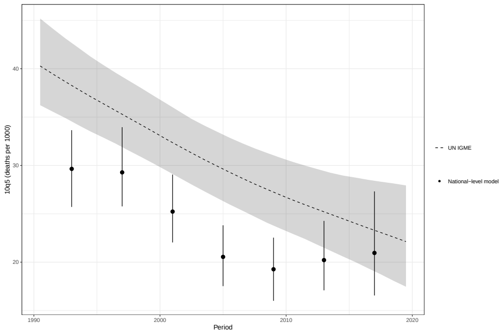

Fig S32. National-level model and UN IGME ${}_{10}q_5$ estimates, Mali

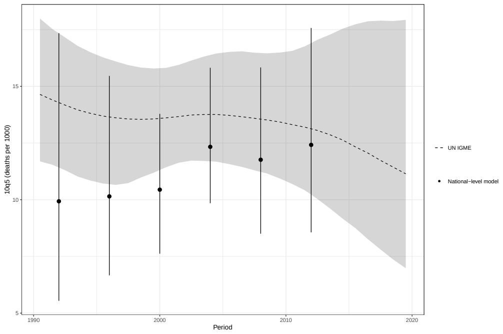

Fig S33. National-level model and UN IGME ${}_{10}q_5$ estimates, Namibia

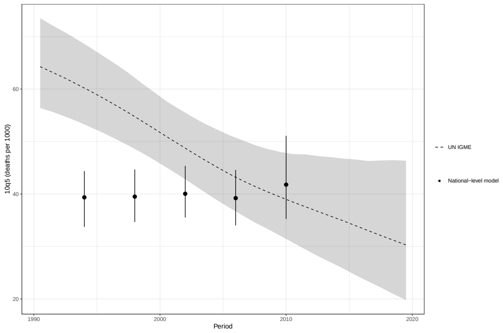

Fig S34. National-level model and UN IGME ${}_{10}q_5$ estimates, Niger

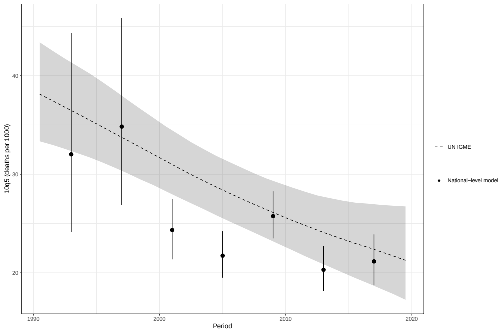

Fig S35. National-level model and UN IGME ${}_{10}q_5$ estimates, Nigeria

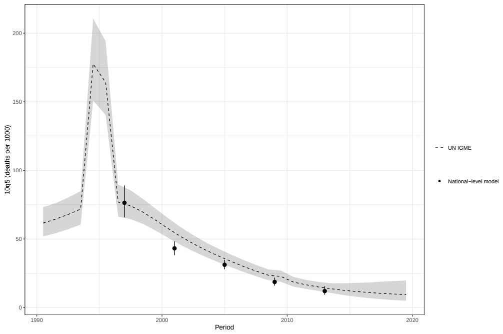

Fig S36. National-level model and UN IGME ${}_{10}q_5$ estimates, Rwanda

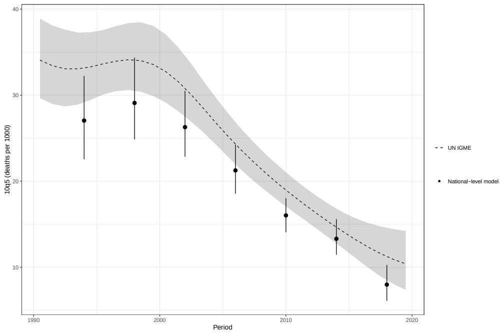

Fig S37. National-level model and UN IGME ${}_{10}q_5$ estimates, Senegal

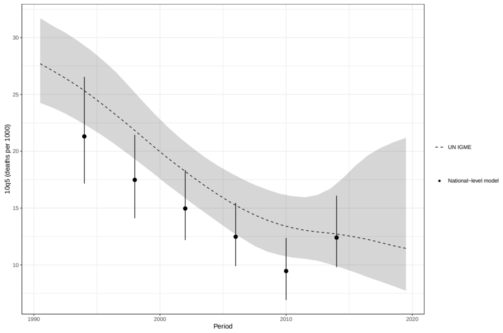

Fig S38. National-level model and UN IGME ${}_{10}q_5$ estimates, Tanzania

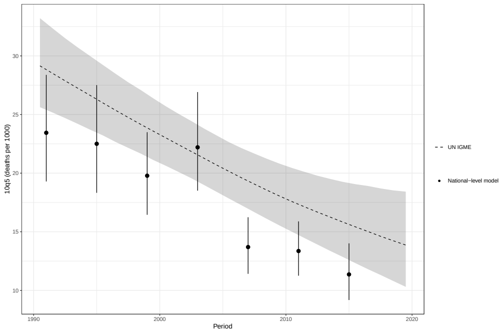

Fig S39. National-level model and UN IGME ${}_{10}q_5$ estimates, Uganda

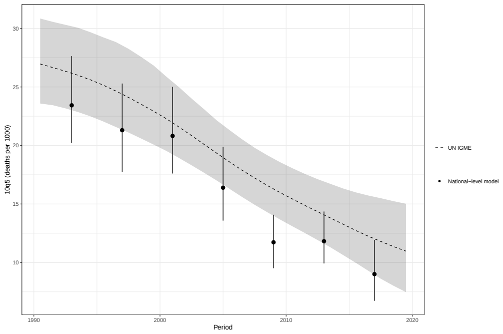

Fig S40. National-level model and UN IGME ${}_{10}q_5$ estimates, Zambia

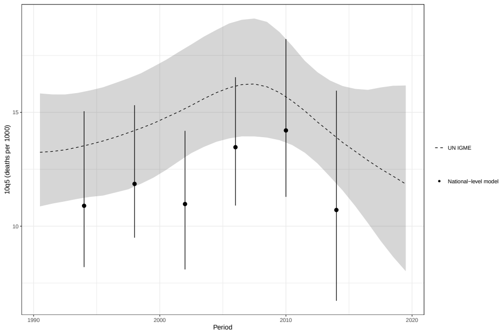

Fig S41. National-level model and UN IGME ${}_{10}q_5$ estimates, Zimbabwe

S5 Sub-national mortality rates

In this section, we compare admin-1 level estimates of the probability of dying ${}_{10}q_5$ with the risk of dying before 5 years of age (${}_5q_0$) for each of the 20 countries in our sample.

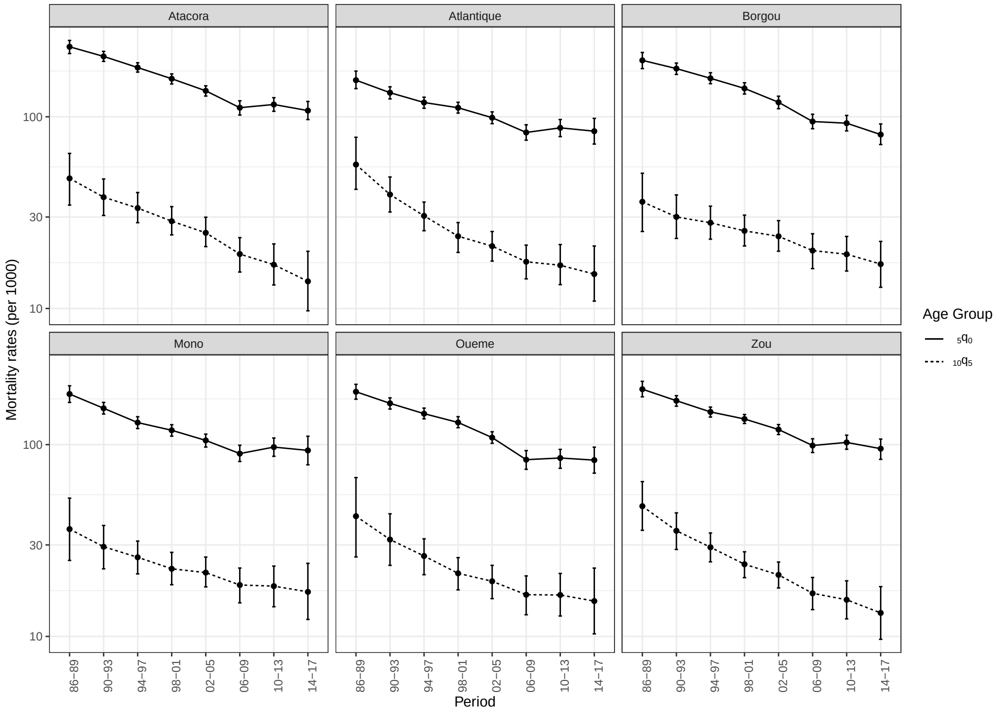

Fig S42. Sub-national mortality rates (log scale), Benin

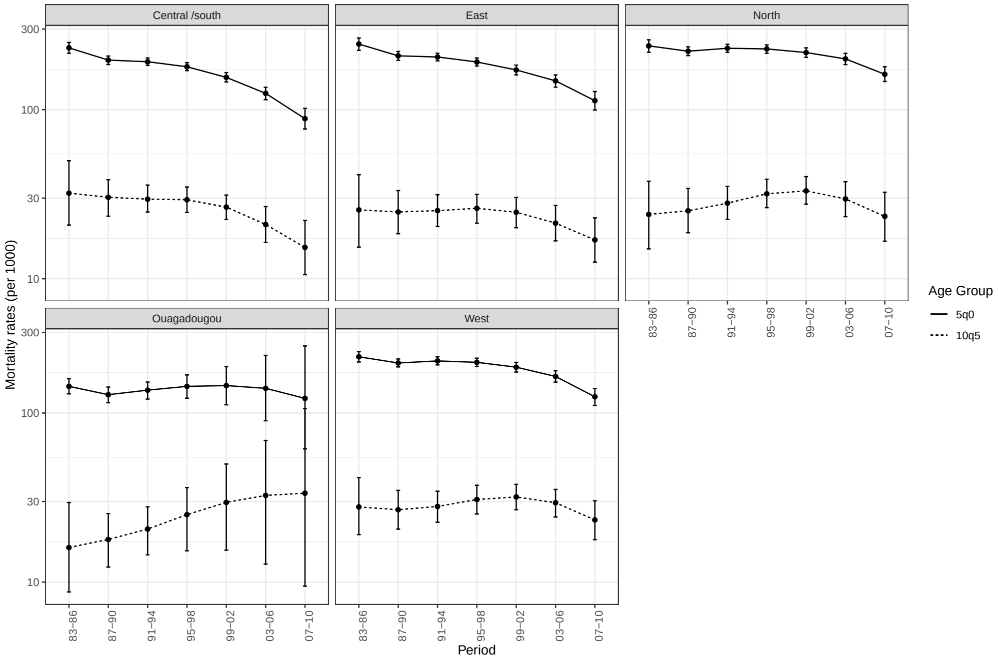

Fig S43. Sub-national mortality rates (log scale), Burkina Faso

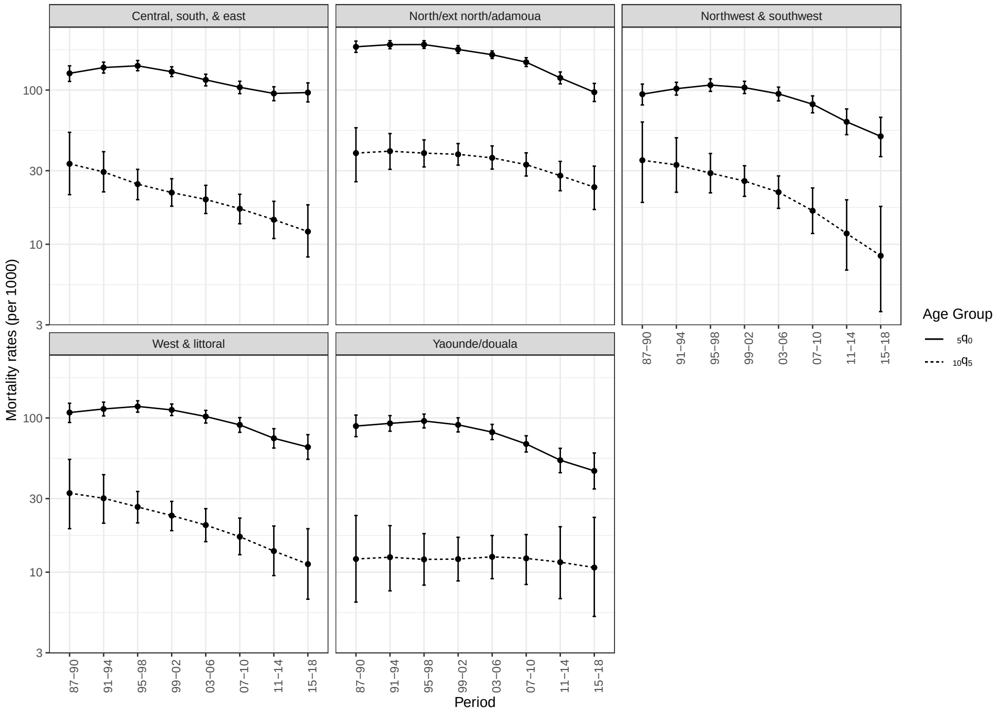

Fig S44. Sub-national mortality rates (log scale), Cameroon

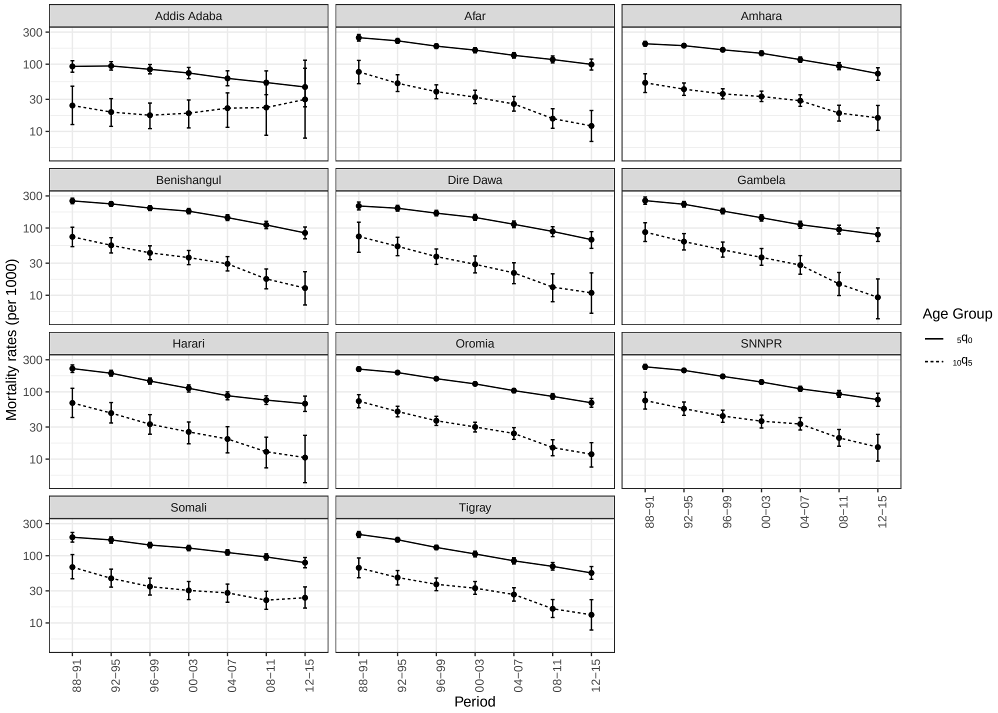

Fig S45. Sub-national mortality rates (log scale), Ethiopia

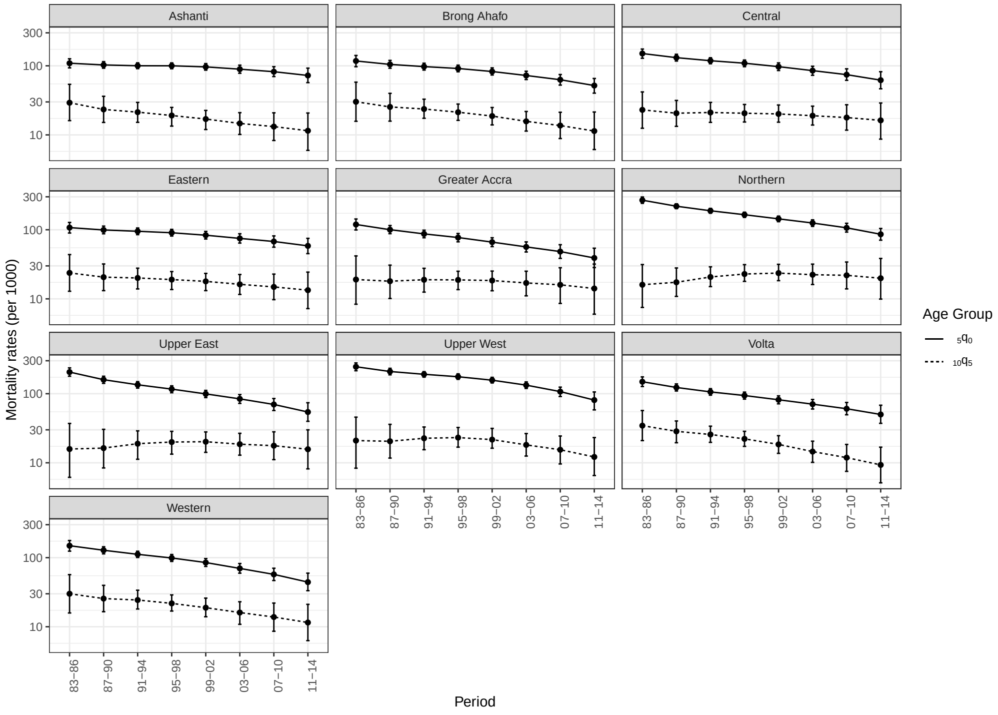

Fig S46. Sub-national mortality rates (log scale), Ghana

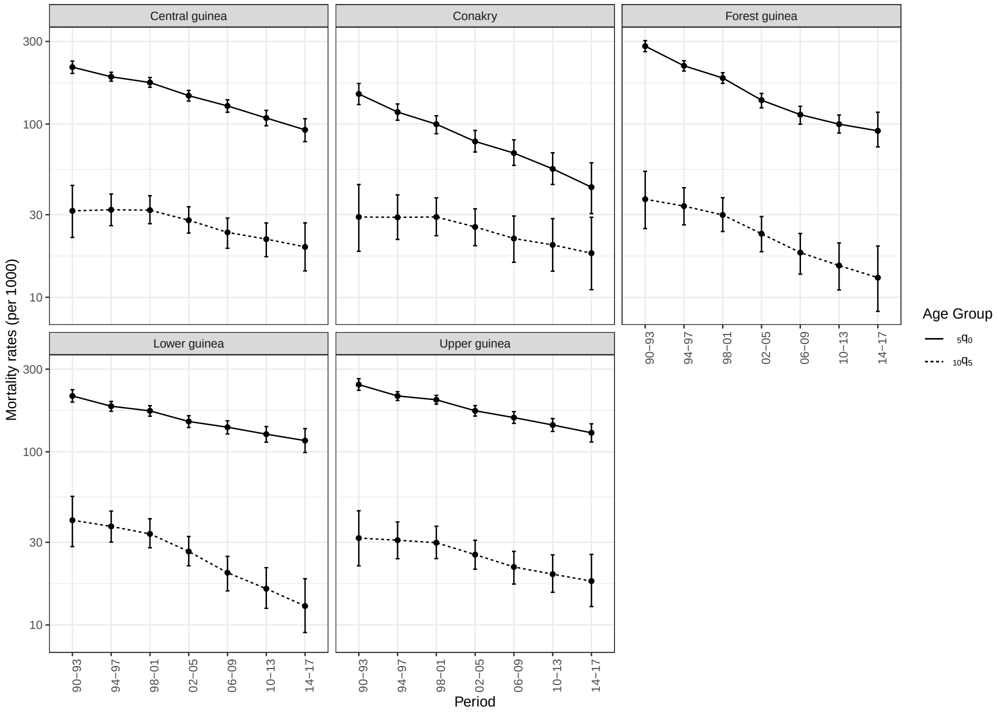

Fig S47. Sub-national mortality rates (log scale), Guinea

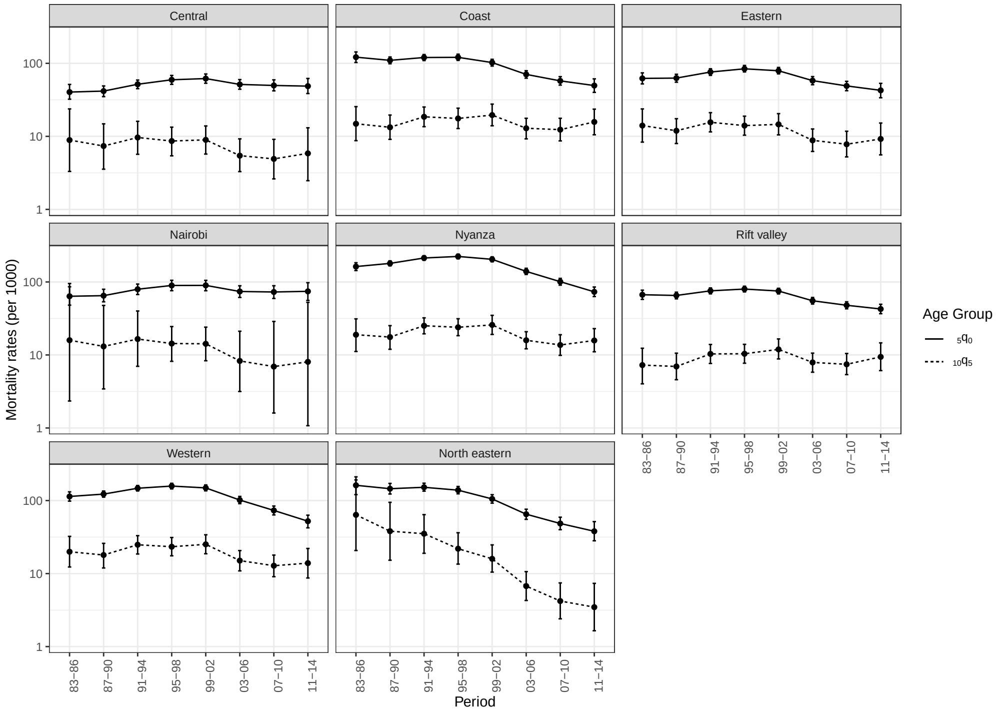

Fig S48. Sub-national mortality rates (log scale), Kenya

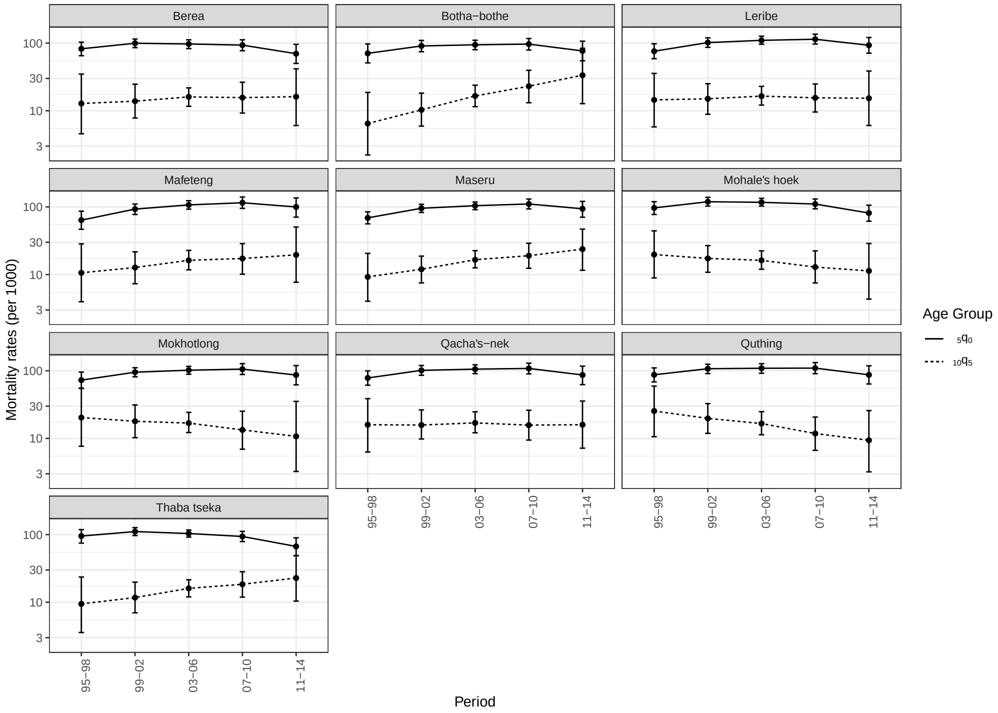

Fig S49. Sub-national mortality rates (log scale), Lesotho

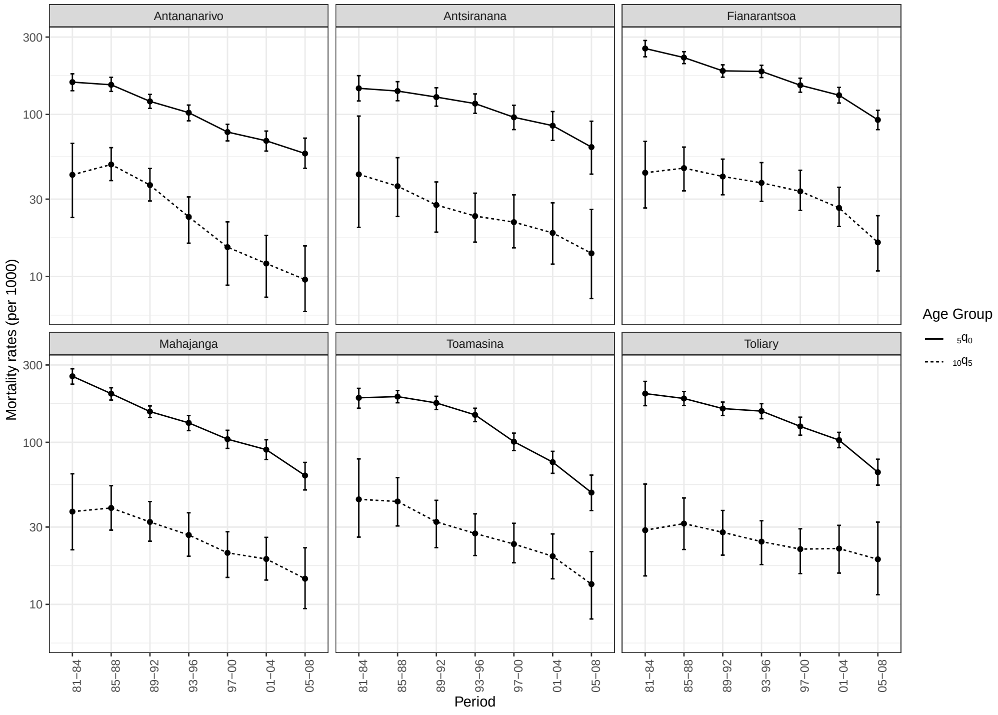

Fig S50. Sub-national mortality rates (log scale), Madagascar

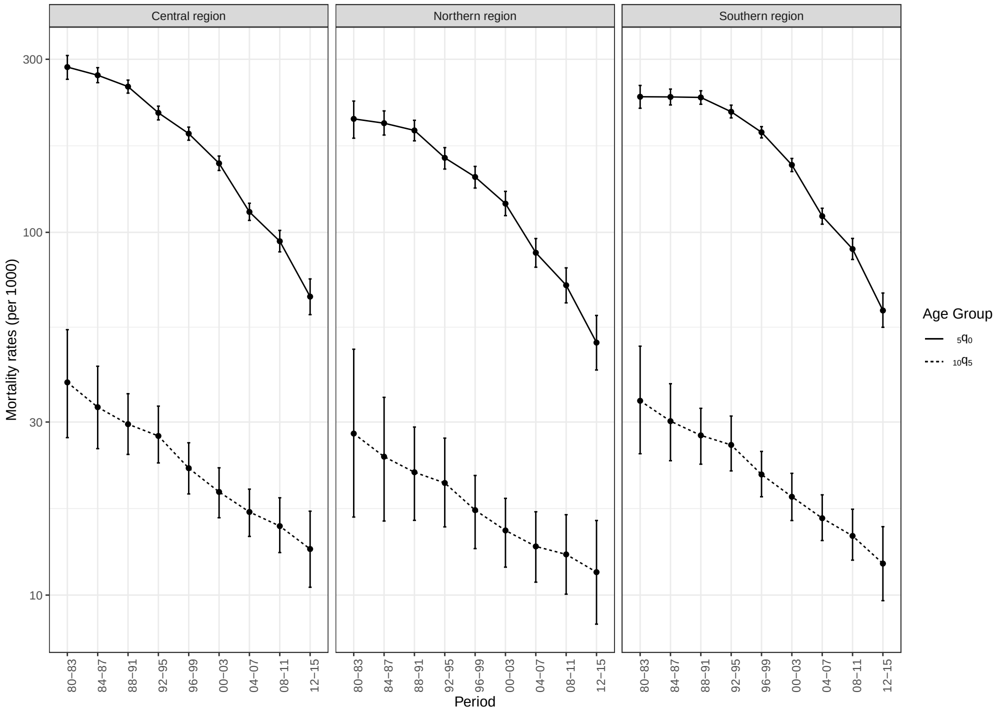

Fig S51. Sub-national mortality rates (log scale), Malawi

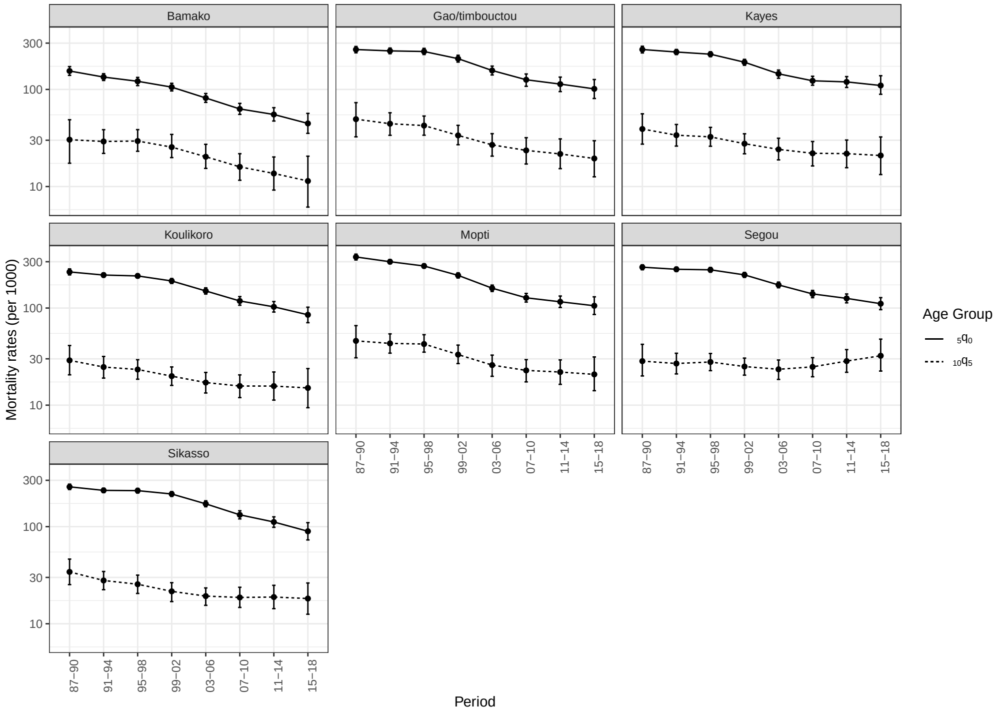

Fig S52. Sub-national mortality rates (log scale), Mali

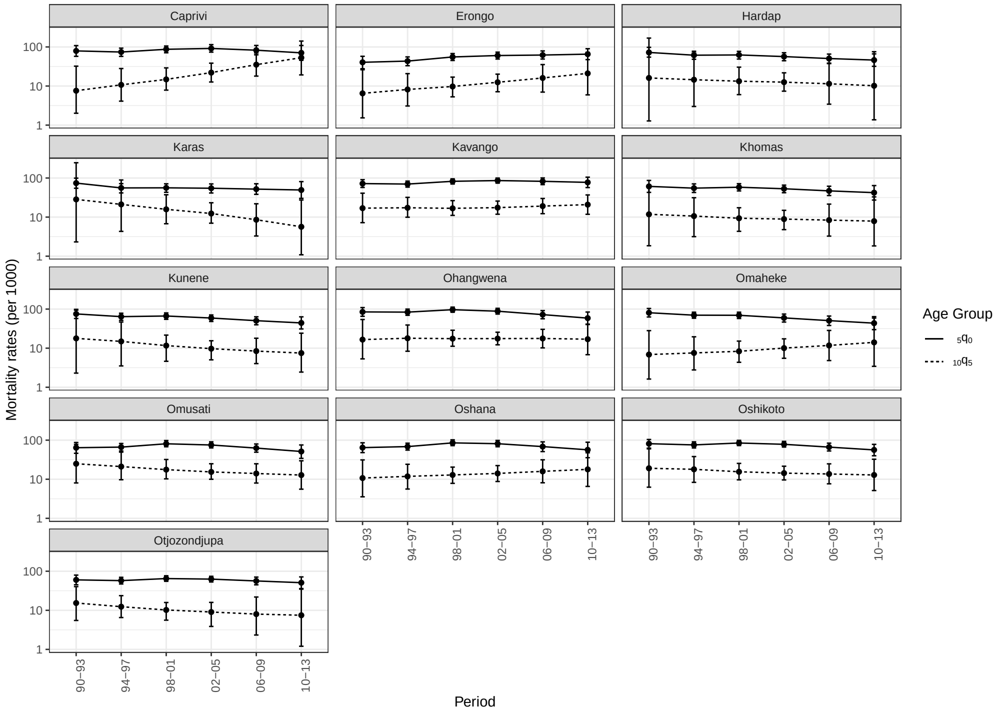

Fig S53. Sub-national mortality rates (log scale), Namibia

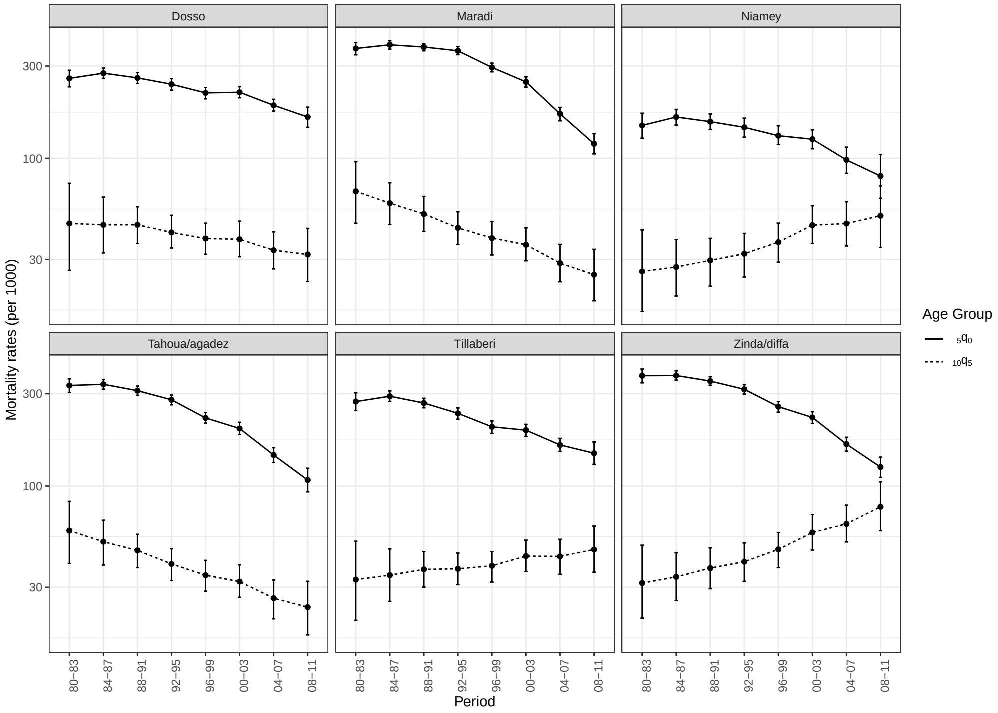

Fig S54. Sub-national mortality rates (log scale), Niger

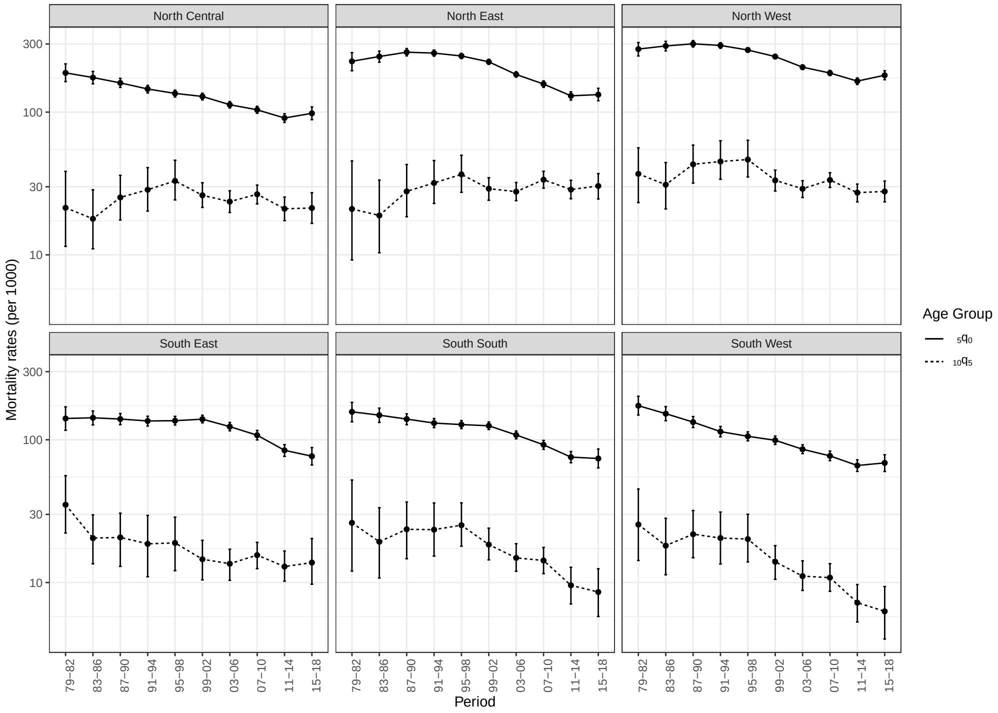

Fig S55. Sub-national mortality rates (log scale), Nigeria

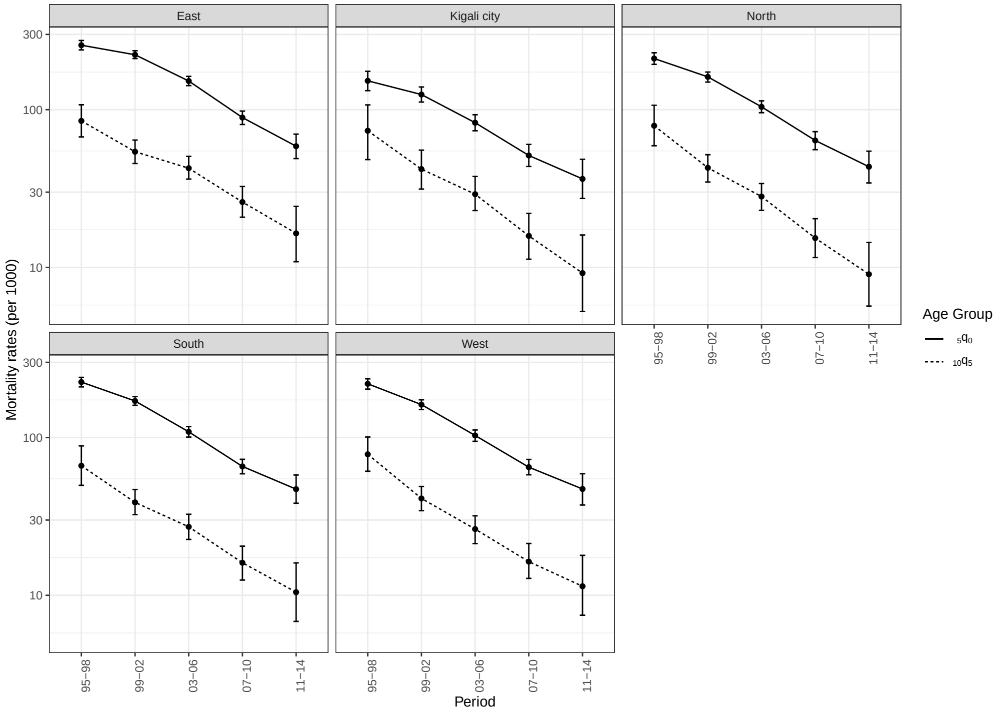

Fig S56. Sub-national mortality rates (log scale), Rwanda

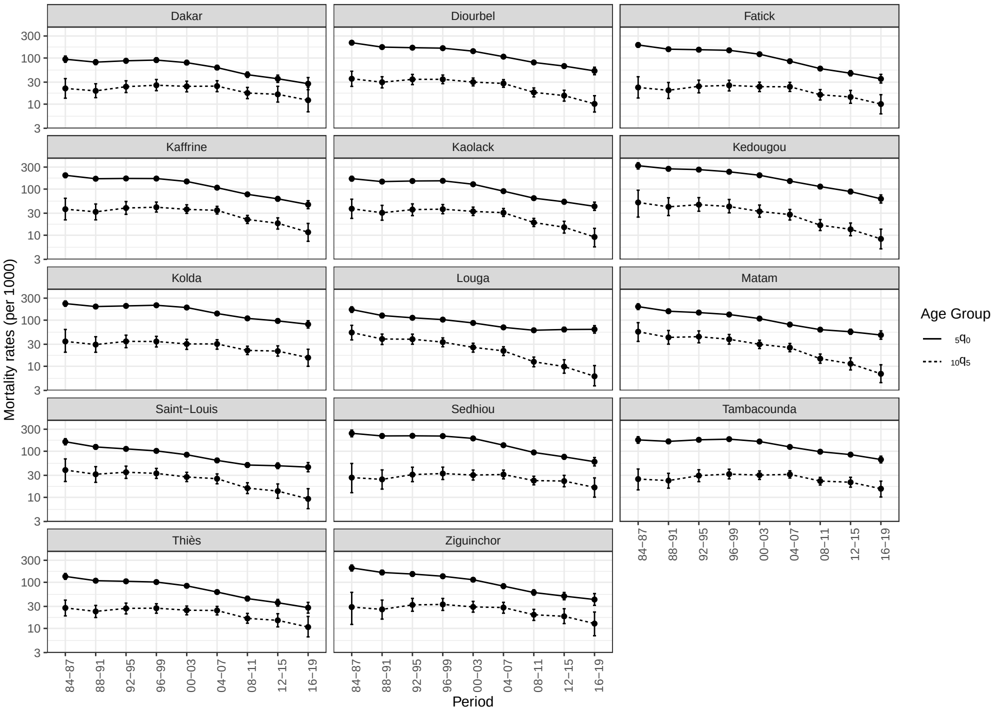

Fig S57. Sub-national mortality rates (log scale), Senegal

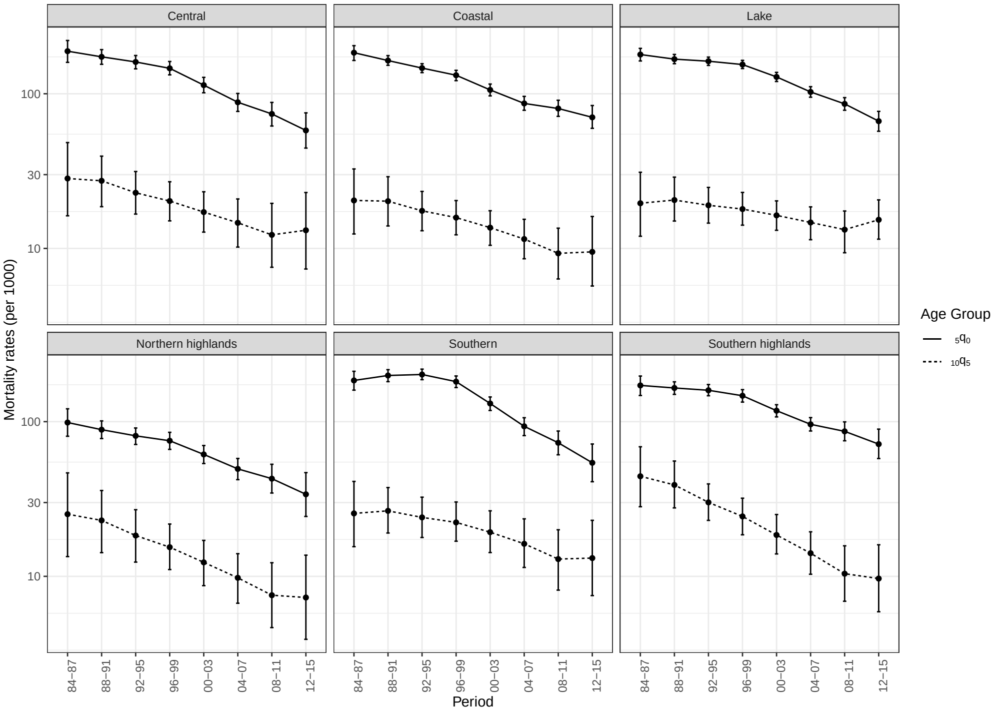

Fig S58. Sub-national mortality rates (log scale), Tanzania

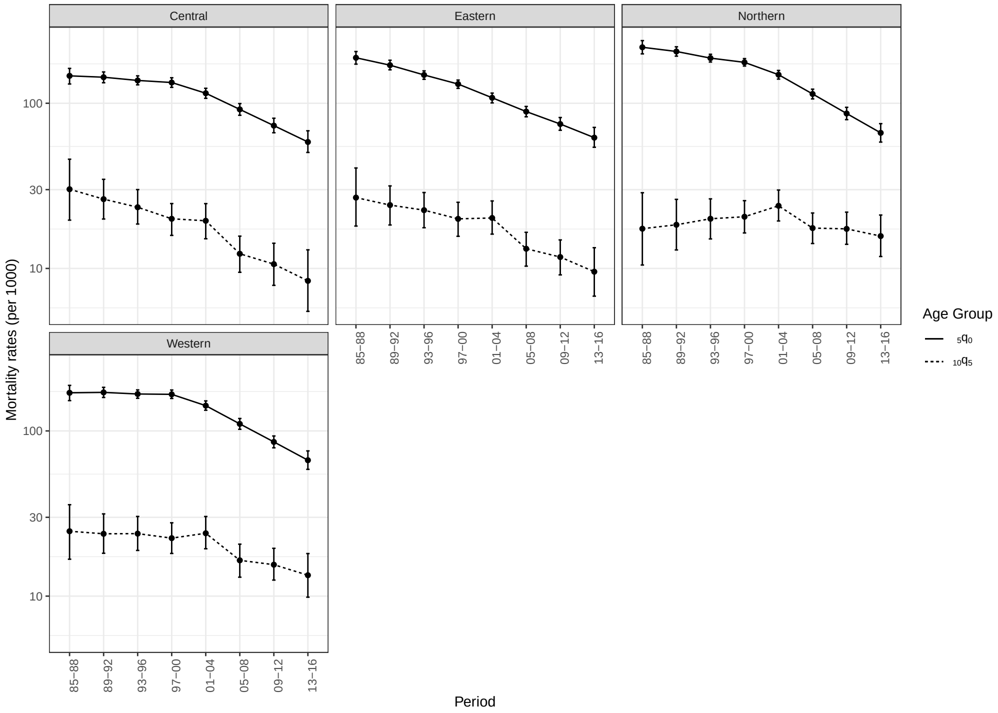

Fig S59. Sub-national mortality rates (log scale), Uganda

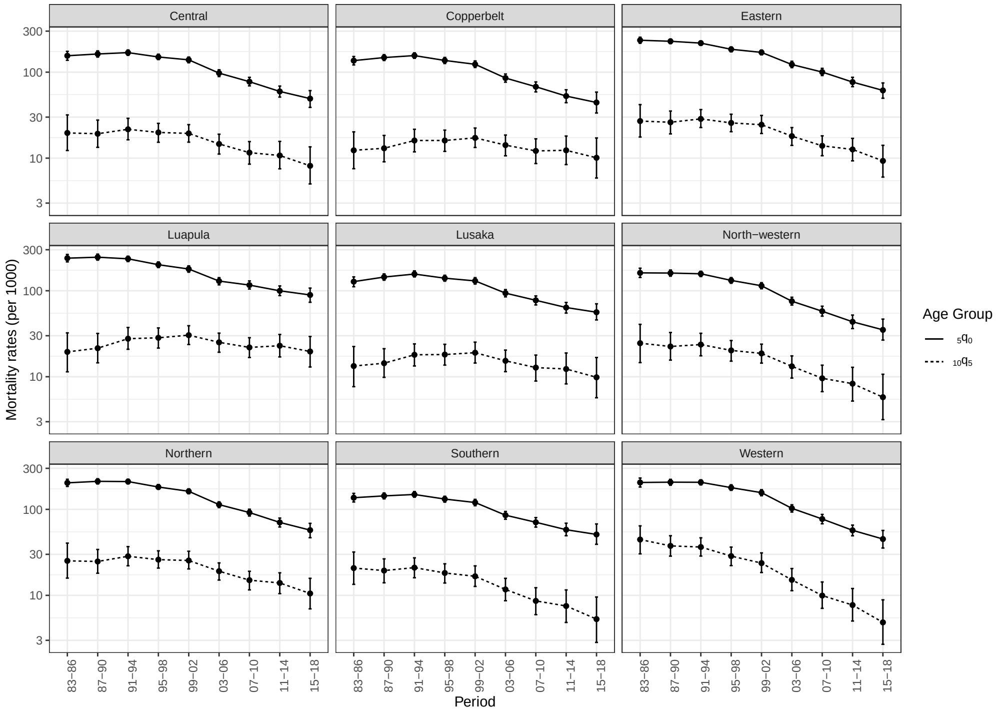

Fig S60. Sub-national mortality rates (log scale), Zambia

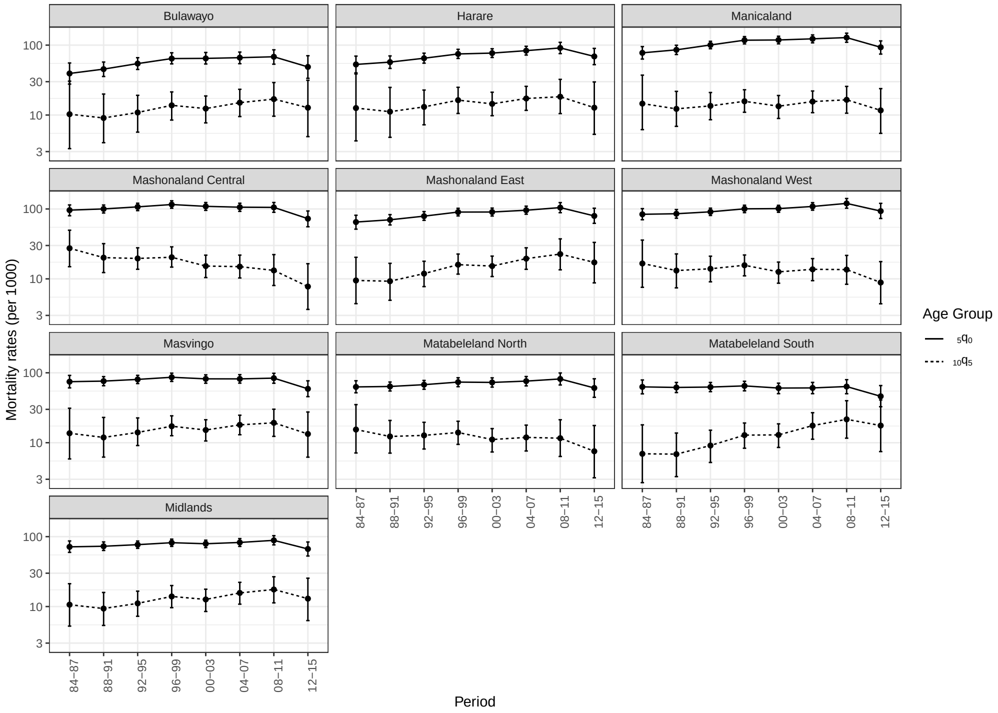

Fig S61. Sub-national mortality rates (log scale), Zimbabwe

S6 Assessment of precision of mortality estimates for children aged less than five years

Figure S62 displays the coefficient of variation for the probability ${}_5\hat{q}_0$ estimated for each Admin 1-period combination. These CVs are compared to the threshold value of 20%. Out of 1,132 space-time estimates, 99.3% fall below the 20% threshold.

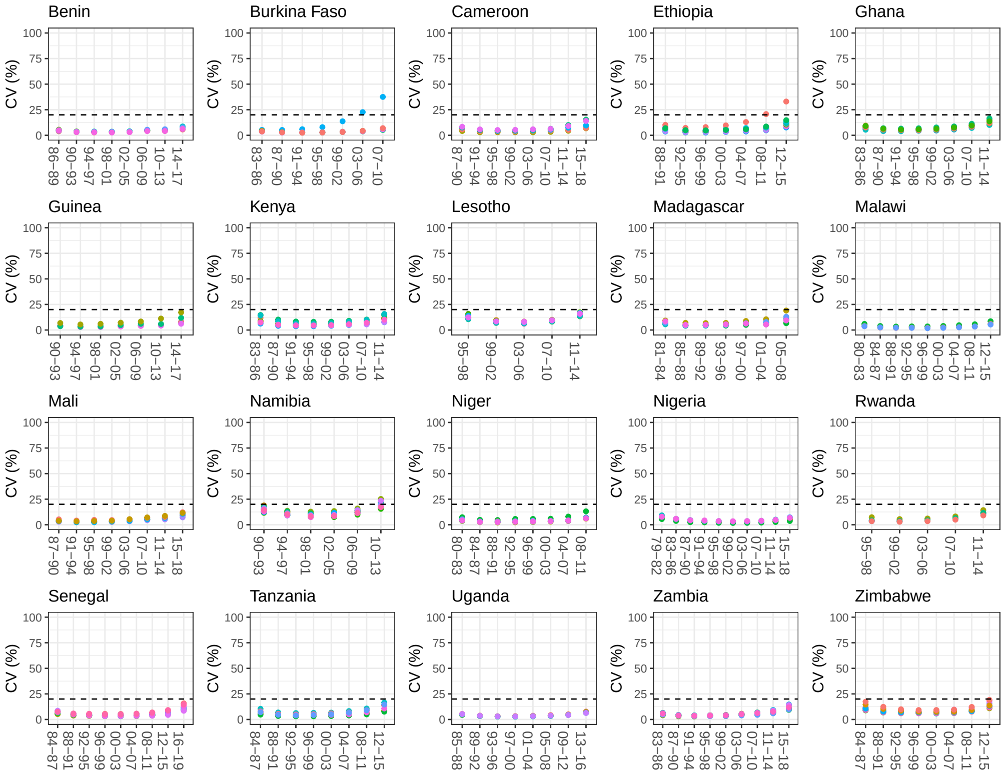

Fig S62. Coefficient of variation associated with sub-national ${}_5q_0$ estimates

S7 Assessment of precision of mortality estimates for children aged 5-14 with a 15% threshold

In Figure S63, we are more conservative and consider periods for which > 75% sub-national mortality estimates of children aged 5-14 were sufficiently precise, where sufficiently precise is now defined as having a ratio between standard deviation and mean below 15%. In this case, out of 1,132 sub-national ${}_{10}\hat{q}_5$ estimates, 37.8% were considered robust. In comparison, this was the case for 95.9% of sub-national ${}_{5}\hat{q}_0$ estimates.

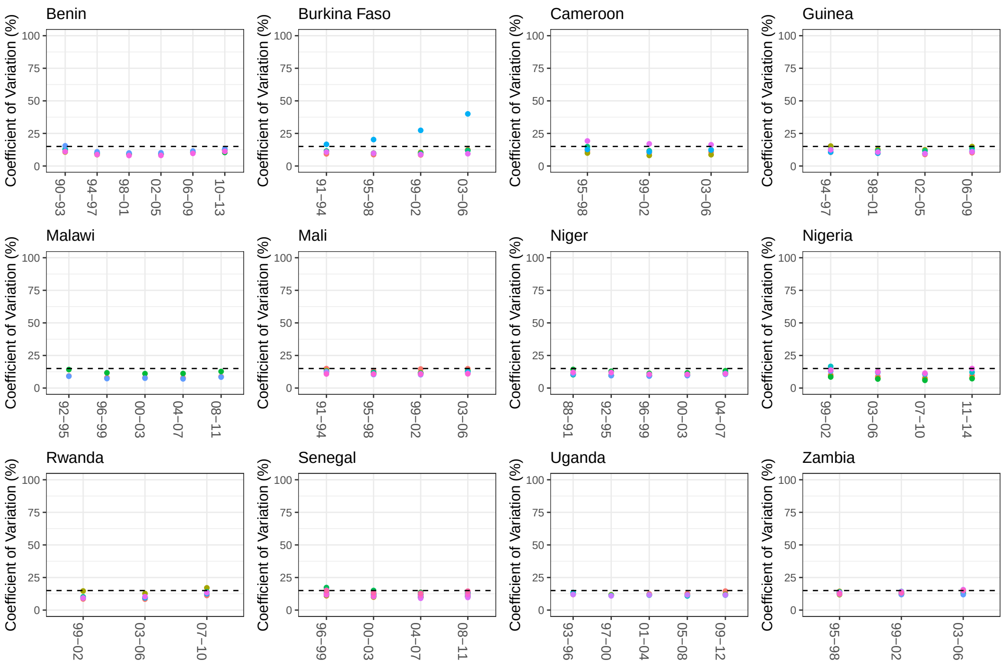

Fig S63. Coefficient of variation associated with precise sub-national ${}_{10}q_5$ estimates (conservative)

S8 Mortality estimates in our sample, compared to all countries in Sub-Saharan Africa

For this analysis, we selected 20 countries out of the 35 countries used by Li and colleagues (2019) (two of which were in Northern Africa). The figure below shows densities of the probability ${}_{10}q_5$ for latest UN IGME estimates associated to both samples of countries. The two samples are similar. When compared to all countries in Sub-Saharan Africa, we note that mortality was slightly higher in our sample. The population-weighted average of the risk of dying in the age group 5-14 was 20 per 1000 in 2019 in these 20 countries, which is slightly higher than the regional estimate for Sub-Saharan Africa (16 per 1000) [2]. In 2019, 65% of all deaths that occurred in Sub-Saharan Africa in the age group 5-14 where in these 20 countries.

Fig S64. Densities and means for ${}_{10}q_5$ estimates by UN IGME for 2019 in the sample of countries retained by Li et al. (2019) and this study

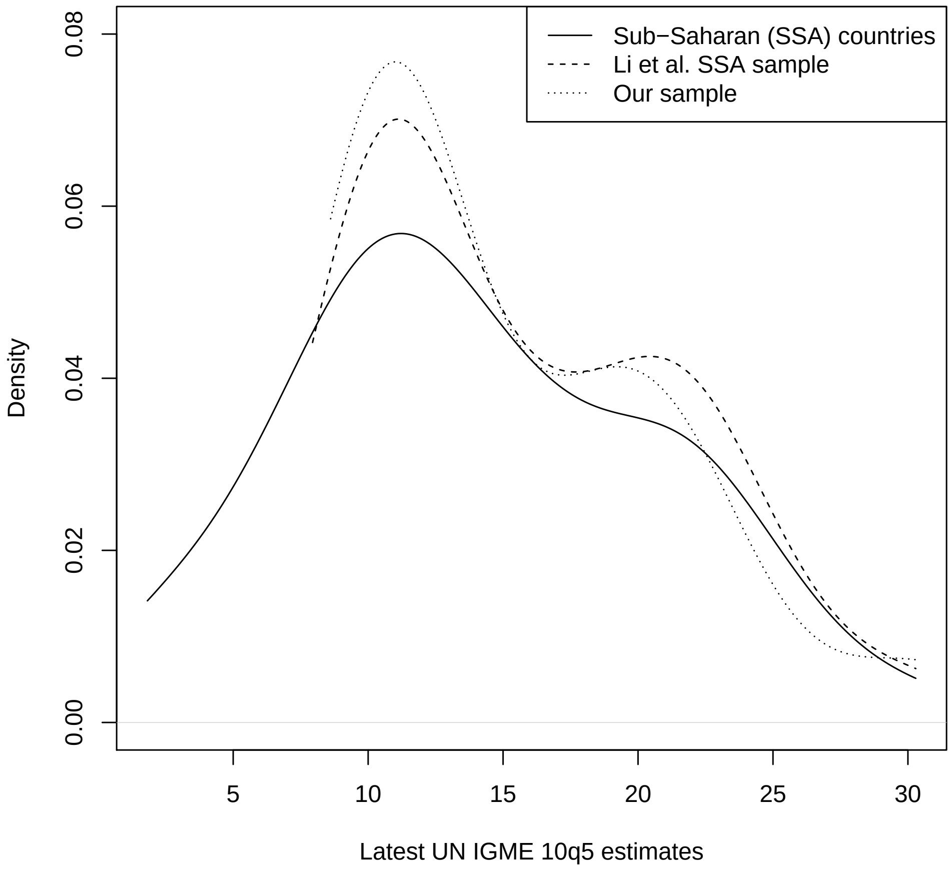

References

1. Alkema L, New JR, Pedersen J, You D. Child mortality estimation 2013: an overview of updates in estimation methods by the United Nations Inter-agency Group for Child Mortality Estimation. PloS one. 2014 Jul 11;9(7):e101112.

2. United Nations Inter-agency Group for Child Mortality Estimation (UN IGME). Levels & Trends in Child Mortality: Report 2020, Estimates developed by the United Nations Inter-agency Group for Child Mortality Estimation. United Nations Children's Fund, New York, 2020.

3. Li Z, Hsiao Y, Godwin J, Martin BD, Wakefield J, Clark SJ, with support from the United Nations Inter-agency Group for Child Mortality Estimation and its technical advisory group. Changes in the spatial distribution of the under-five mortality rate: Small-area analysis of 122 DHS surveys in 262 subregions of 35 countries in Africa. PloS one. 2019 Jan 22;14(1):e0210645.

4. Masquelier B, Hug L, Sharrow D, You D, Hogan D, Hill K, Liu J, Pedersen J, Alkema L. Global, regional, and national mortality trends in older children and young adolescents (5–14 years) from 1990 to 2016: an analysis of empirical data. The Lancet Global Health. 2018 Oct 1;6(10):e1087-99.
